# Multi-Color Single Particle Tracking with Quantum Dots

**DOI:** 10.1371/journal.pone.0048521

**Published:** 2012-11-14

**Authors:** Eva C. Arnspang, Jonathan R. Brewer, B. Christoffer Lagerholm

**Affiliations:** 1 Department of Physics, Chemistry and Pharmacy, University of Southern Denmark, Odense M, Denmark; 2 Department of Biochemistry and Molecular Biology, University of Southern Denmark, Odense M, Denmark; 3 MEMPHYS – Center for Biomembrane Physics, and DaMBIC – Danish Molecular Biomedical Imaging Center, University of Southern Denmark, Odense M, Denmark; Aligarh Muslim University, India

## Abstract

Quantum dots (QDs) have long promised to revolutionize fluorescence detection to include even applications requiring simultaneous multi-species detection at single molecule sensitivity. Despite the early promise, the unique optical properties of QDs have not yet been fully exploited in e. g. multiplex single molecule sensitivity applications such as single particle tracking (SPT). In order to fully optimize single molecule multiplex application with QDs, we have in this work performed a comprehensive quantitative investigation of the fluorescence intensities, fluorescence intensity fluctuations, and hydrodynamic radii of eight types of commercially available water soluble QDs. In this study, we show that the fluorescence intensity of CdSe core QDs increases as the emission of the QDs shifts towards the red but that hybrid CdSe/CdTe core QDs are less bright than the furthest red-shifted CdSe QDs. We further show that there is only a small size advantage in using blue-shifted QDs in biological applications because of the additional size of the water-stabilizing surface coat. Extending previous work, we finally also show that parallel four color multicolor (MC)-SPT with QDs is possible at an image acquisition rate of at least 25 Hz. We demonstrate the technique by measuring the lateral dynamics of a lipid, biotin-cap-DPPE, in the cellular plasma membrane of live cells using four different colors of QDs; QD565, QD605, QD655, and QD705 as labels.

## Introduction

The last two decades have seen a dramatic increase in the utilization of single molecule detection methods in a variety of biological applications [1]–[4]. For example, the single molecule method SPT has revealed new dynamic information about the molecular organization of the cellular plasma membrane [1], [5]. But spatio-temporal investigations using SPT are particular prone to artifacts due to e.g. the probe size and valency [6]–[8]. This is because SPT has historically been performed with antibody conjugated colloidal gold particles [9], [10] that are large (R_H_≈20–25 nm) and for which the binding valency towards the target molecule is challenging to control and validate [8]. Colloidal gold particles are furthermore detected by Rayleigh light scattering which precludes their use in multiplex applications.

More recently quantum dots (QDs) have seen increased use in SPT [1], [11]–[17]. QDs, which are colloidal semiconductor fluorescent nanocrystals, are perhaps the best example of the exceptional advantages that nanotechnology can provide by enabling the synthesis of materials with significantly enhanced properties as compared to conventional materials. Importantly for use in SPT, the fluorescence intensities of QDs are exceptionally large due to very large absorption cross-sections, where the larger the QD core-shell, the greater the absorption cross-section, and that QDs can be manufactured with close to ideal quantum yields (QY) [18]. The emission spectrum of QDs is furthermore tunable and depends on the core-shell diameter and the composition. QDs are consequently available over a wide range of emission wavelengths, from the lower visible spectra to the near-infrared. QDs also have wide overlapping excitation spectra yet narrow symmetric emission spectra making them ideal for multiplex applications [19] to include also SPT but only for up to two colors [11], [16], [20]. Five color QD labeling of the same species has also been claimed but no results that such labeling and foremost that detection was achieved at the single QD level has yet been presented [12]. The optical properties of QDs are however not perfect as both blinking and bleaching is possible even though the impact of both of these processes can be minimized (but not prevented) by use of small reducing agents such as β-mercaptoethanol (β-Me) and dithiothreitol (DTTs) [21], [22] [23]. Non-blinking QDs have also been reported [24],but these are not applicable for use in MC-SPT because of their broad spectral emission bandwidth (>100 nm). Available commercial QDs are also not optimized for monovalent binding towards their target [25] although custom conjugated monovalent QDs have been reported [20], [26], [27].

The objective of this work was to extend parallel SPT with QDs from two to four colors. In order to achieve this, we have performed a comprehensive quantitative comparison of the fluorescence intensities, fluorescent intensity fluctuations and size of eight types of QDs (QD525, QD565, QD585, QD605, QD625, QD655, QD705, and QD800). We have used single molecule fluorescence imaging in order to quantify the fluorescence intensity and intensity fluctuations of these QDs. We have furthermore used fluorescence correlation spectroscopy (FCS) to determine the hydrodynamic radii (R_H_) of all but the QD800s. Based on these results we have designed and assembled a fluorescence microscope capable of parallel four color SPT using QD565, QD605, QD655 and QD705. Finally, we demonstrate an application of parallel four color MC-SPT by investigating the lateral dynamics of an artificial lipid, biotin-cap-DPPE in the plasma membrane of a live mouse embryo fibroblast (MEFs).

## Results and Discussion

### Fluorescence Intensities and Intensity Fluctuations of Single QDs

**Figure 1:**
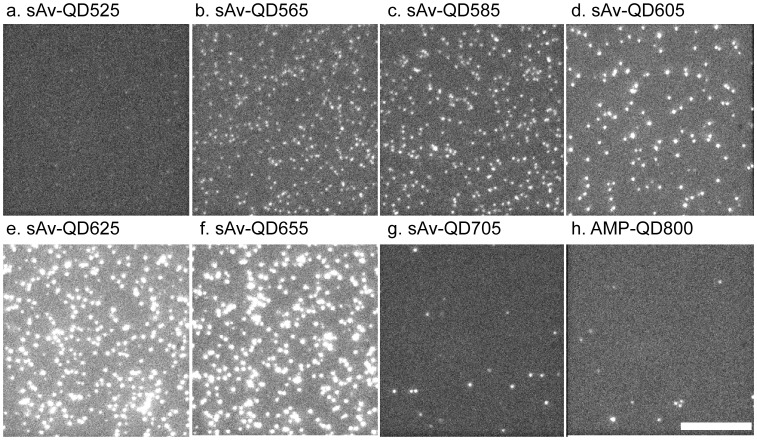
QD Representative single image frame comparison of the fluorescence intensities of QD525, QD565, QD585, QD605, QD625, QD655, QD705, and QD800. All images are displayed at identical brightness and contrast ratios and are directly comparable. The entire image sequence is shown in Movie S1. Scale bar equals 10 µm.

In order to optimize the performance of MC-SPT, we first quantified the fluorescence intensity and intensity fluctuations of the QDs by single molecule fluorescence imaging. The samples in these experiments consisted of QDs that had been non-specifically adsorbed to a glass coverslip at coating densities such that a large majority of observed QDs were identified as single QDs as has been described before [28]. A representative image, from a set of image sequences for each QD type (Movie S1) in which each image set was acquired under equivalent conditions is shown in Fig. 1.

**Figure 2:**
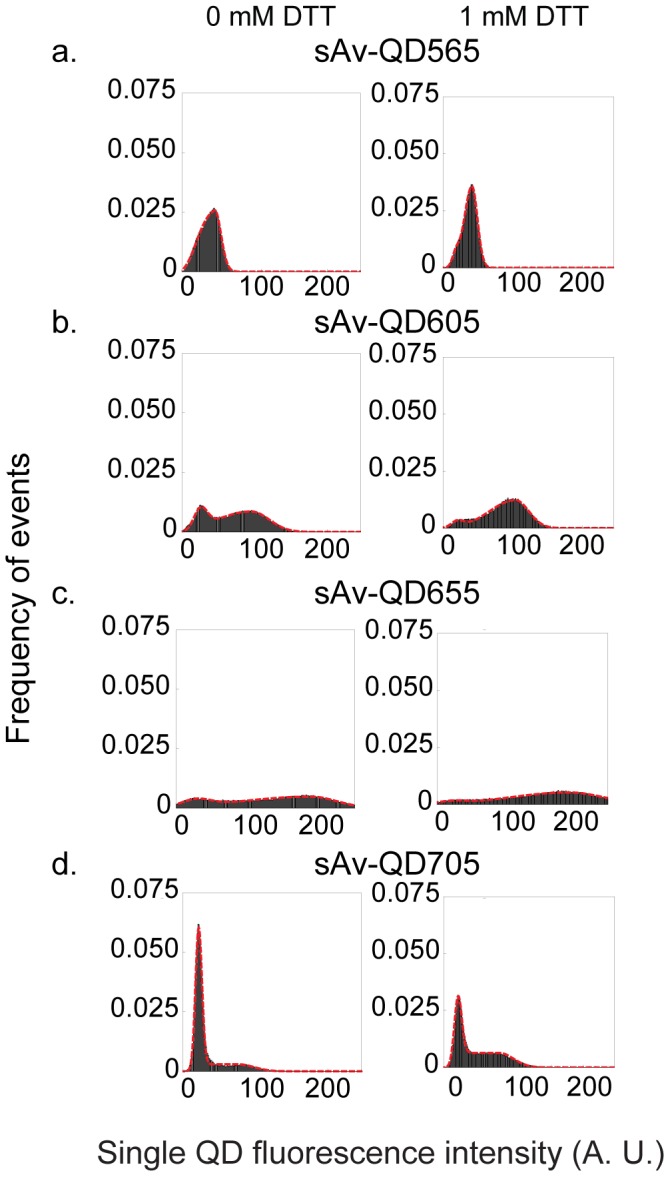
Cumulative frequency histograms of the background subtracted fluorescence intensities per pixel of 9×9 pixel arrays of identified single sAv-QD565, sAv-QD605, sAv-QD655, and sAv-QD705 in absence of DTT (left), and in presence of 1 mM DTT (right). These histograms were generated from 3 fields of view in total containing between ∼100≤n≤∼600 single QDs that were each imaged for m = 300 image frames. The total sampling points for each histogram (m n) were ∼30,000≤m n≤∼180,000. The mean fluorescence intensities, I_QD_^on^, and fractional “on” time, F_QD_^on^, were determined by non-linear curve fitting (red dashed line) as described in the Methods section. The results for sAv-QD525, sAv-QD585, sAv-QD625, and AMP-QD800 are given in Fig. S1.

**Figure 3:**
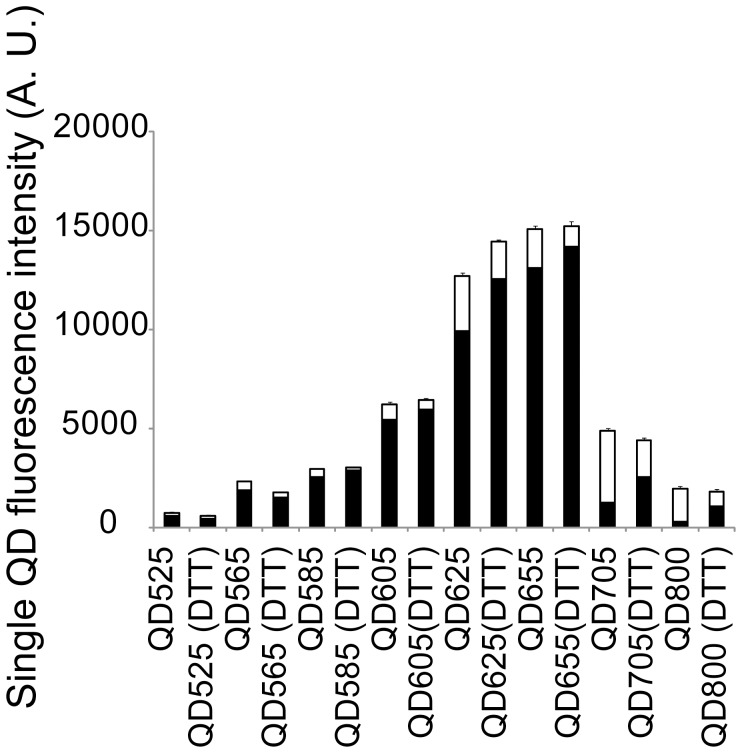
Mean fluorescence intensities and fractional “on” time of single QDs showing the ensemble average mean (+s.e.m) integrated fluorescence intensity, I_QD_^on^, (full bar) and fractional on (filled bar)/off (unfilled bar) times, F_QD_^on/off^ of all QD colors with and without 1 mM DTT. A summary of the values for all QD colors and conditions is given in Table S2.

The acquired image sequences were subjected to image analysis by use of a combination of ImageJ and Mathematica in order to quantitatively analyze the differences in fluorescence intensity and intermittency of the different colors of single QDs as has been described before [28]. The analyzed data was from a minimum of three fields of view with a range of total identified single QDs from ∼100 to >500, each for 300 sampling points, corresponding to a range of ∼30,000 to ∼200,000 sample points. From this data, we subsequently generated cumulative frequency histograms of the fluorescence intensities from the time evolution of all single QDs that were identified for a given QD color and experimental condition (±1 mM DTT). These intensity histograms were curve fit to yield quantitative results of the ensemble average mean fluorescence intensity, I_QD_^on^, and fractional “on” time, F_QD_^on^, for each color of QD in each condition (Fig. 2 and Fig. S1). The results for I_QD_^on^ and F_QD_^on^ for each QD color and experimental condition are summarized in Fig. 3 and in detail in Table S2.The results show that there is quantitative difference in I_QD_^on^ among the different colors of QDs where the order, ranked in order of decreasing I_QD_^on^, was I_QD625_^on^≈I_QD655_^ on^>I_QD605_^on^≈I_QD705_^ on^>I_QD565_^ on^≈I_QD585_^ on^≈I_QD800_^on^>I_QD525_^ on^. In these results, we have not accounted for the spectral dependence of the quantum efficiency (QE) of the CCD camera (Fig. S2) which in this case would result in an increase of the absolute brightness of the QD705s by ≈5% and of QD800s by ≈25%. The trend of the fluorescence intensity of the QDs is hence as expected that the fluorescence intensity increases in the direction of increasing core size, from QD525 to QD655. But there is a decrease in the fluorescence intensity between QD655 and QD705 and QD800. This is most likely a consequence of the changed composition of the core/shell materials of these QDs which according to the manufacturer is CdSe/ZnS for QD525 to QD655 and CdSeTe/ZnS for QD705 and QD800.

These results also show that there is a quantitative difference in the fractional “on” time, F_QD_^on^, among the different colors of QDs where the order, ranked in order of decreasing F_QD_^on^, was F_QD525_^on^≈F_QD565_^on^≈F_QD585_^on^≈F_QD605_^on^≈F_QD625_^on^≈F_QD655_^on^>>>F_QD705_^on^≈F_QD800_^on^. It is especially noteworthy that under the imaging conditions used in this comparison the near infrared QDs, QD705 (∼70% of time in “off” state) and QD800 (∼85% of time in “off” state) spend a majority of their time in a dark non-fluorescent state while the remaining QDs are principally in their bright fluorescent “on” state. Again, this is likely a consequence of the changed composition of the core/shell materials of these QDs.

**Figure 4:**
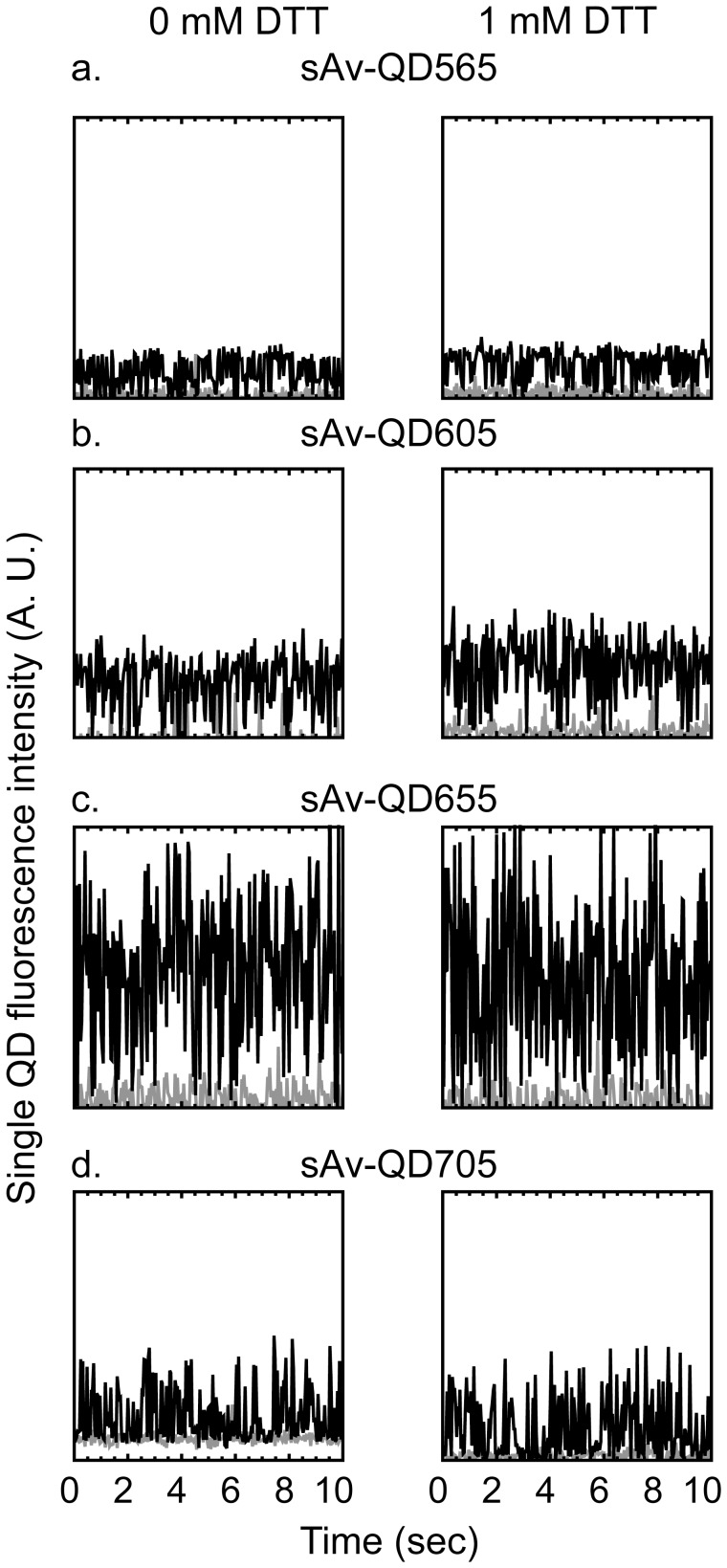
Representative, background subtracted, integrated fluorescence intensities, as a function of time, of identified single sAv-QD565, sAv-QD605, sAv-QD655, and sAv-QD705 (black lines) that had been absorbed non-specifically to a glass coverslip in the absence of DTT (left) and in the presence of 1 mM DTT (right). Images of QDs with different peak emissions were acquired under identical conditions with continuous blue illumination with 5 ms camera integration at an image acquisition rate of ≈25 Hz for 300 image frames. Also shown are the fluorescence intensity fluctuations of the background (grey lines). The results for sAv-QD525, sAv-QD585, sAv-QD625, and AMP-QD800 are given in Fig. S3.

Representative examples from this analysis of the temporal fluctuations of the fluorescence intensity of thus identified single QDs for each QD color, ±1 mM DTT are shown in Fig. 4 (and Fig. S3). These examples show, consistent with previous results, that the fluorescence intensity of single QDs in each case fluctuates from a dark “off” state to a bright fluorescent “on” state and that the frequency of these fluctuations changes in the presence of DTT [21], [22]. Similar results are also seen in the presence of 1 mM β-ME.

### Hydrodynamic Radii of QDs

**Figure 5:**
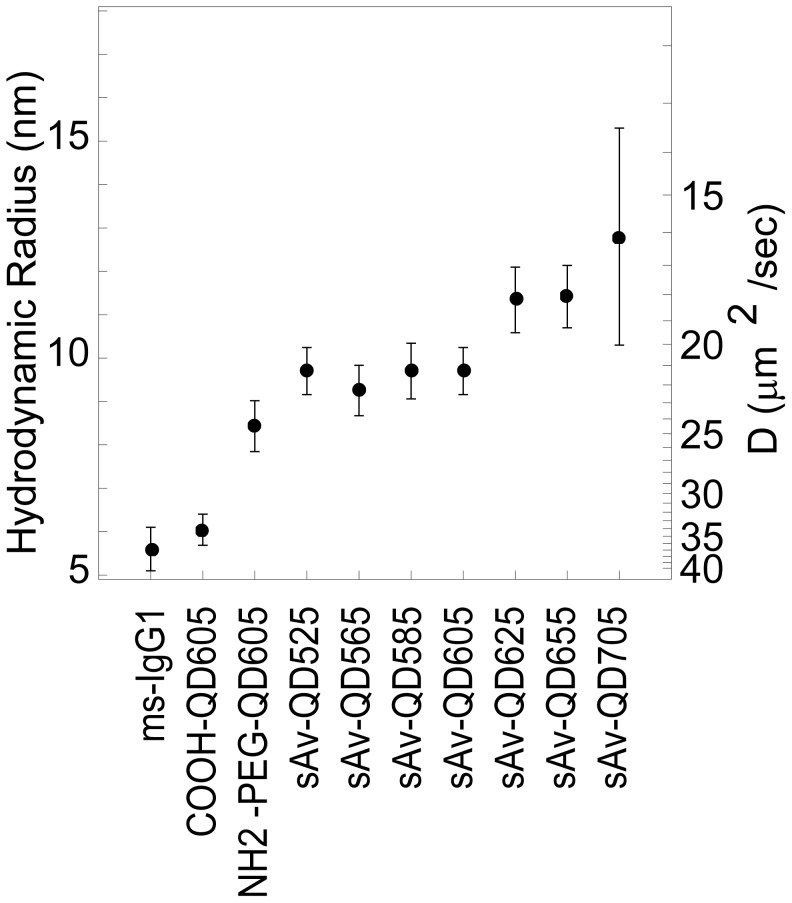
The diffusion coefficient, D, of commercially available QDs in aqueous buffer was measured with FCS. The hydrodynamic radius, R_H_, was estimated from the Stokes Einstein relation with T = 293 K and η = 1.04****cP. The calculated R_H_ are given in Table S3.

The sizes of the QD materials were determined by FCS (Fig. 5 and Table S3). These measurements show that the basic amphiphillic (AMP) water soluble QD605s (AMP-QD605) are similar in size (R_H_≈6 nm) to a fluorescently labeled mouse IgG1 [29]. Non-targeted QDs are also available with an additional amino-PEG coating which adds an additional ≈2.5 nm to the R_H_. Targeted QDs are available as either chemical conjugates to the basic AMP coating or to the additional amino-PEG coating. In this case, we used QDs that had sAv conjugated directly to the basic AMP coating. The R_H_ of these materials is approximately independent of the QD core-shell where sAv-QD525, sAv-QD565, sAv-QD585, and sAv-QD605 are ≈9.5 nm or ≈1.7× the size of a mouse IgG1, sAv-QD625 and sAv-QD655 are ≈11.5 nm or ≈2× the size of a mouse IgG1, and sAv-QD705 are ≈13 nm or ≈2.3× the size of a mouse IgG1 (Fig. 5). These results indicate that there is only a minimal size gain advantage in using water-stabilized QDs with smaller core-shell sizes.

### Localization Precision of SPT with QDs

The localization precision of SPT with QDs was determined by image analysis of time-lapse sequences of immobilized QDs on glass with 5****ms camera integration (Fig. S4 and S5). The precision, δr, was found to be sAv-QD525: δr≈30 nm (N = 254), sAv-QD565: δr≈22 nm (N = 1441), sAv-QD585: δr≈22 nm (N = 1187), sAv-QD605: δr≈14 nm (N = 8598), sAv-QD625: δr≈16 nm (N = 1155), sAv-QD655: δr≈14 nm (N = 2979), sAv-QD705: δr≈16 nm (N = 427), and AMP-QD800: δr≈23 nm (N = 337), where the number in parenthesis denotes the total number of measurements in each case. A plot of the localization precision as a function of the I_QD_^on^ further indicates that the maximum achievable localization precision in the current set-up is δr≈14 nm, corresponding to a precision of ±10 nm along either the x- or y- axis (Fig. S5).

### Multi-color SPT with QDs

Based on these results as well as the spectral separation of the available QDs, we have designed an appropriate fluorescence dichromatic mirror and filter combination to enable parallel four color MC-SPT with QD565, QD605, QD655, and QD705, on a single camera by use of a QuadView image splitter. In making this choice of QDs, we had to make a compromise for the low fractional “on” time, F_QD_^on^, of QD705 in exchange of decreased spectral overlap between adjacent fluorescence channels. The filters chosen for these experiments are listed in the Methods section and the spectra are shown in Fig. S2. Noteworthy is that we typically use a wider bandpass filter for detection of QD565 (40****nm bandwidth compared to 20 nm for QD605 and QD655), in order to account for the observed differences in fluorescence intensity between, in particular, QD565 compared to QD655. We typically also don’t use a fluorescence emission filter in the fluorescence channel for detecting QD705, however, the fluorescence signal in all cases is pre-filtered with a 500 nm longpass fluorescence emission filter (Fig. S2).

The fluorescence intensity of sAv-QD565, sAv-QD605, sAv-QD655, and sAv-QD705 in the QuadView microscope filter configuration (Fig. S2) was determined as before (Figs. S6 and S7 and Table S4). The order of ensemble average mean fluorescence intensity, I_QD_^on^_,_with the restrictions that the additional fluorescence filters place ranked in order of decreasing I_QD_^on^, was I_QD605_^on^>I_QD655_^ on^>I_QD705_^ on^>I_QD565_^ on^. At these settings and with 10 ms integration time, the localization precision, δr, of the MC-SPT was found to be for sAv-QD565: δr≈21 nm (N = 1028), sAv-QD605: δr≈14 nm (N = 1637), sAv-QD655: δr≈15 nm (N = 2689), and sAv-QD705: δr≈28 nm (N = 514) (Table S5 and Fig. S9). We have also evaluated the spectral overlap of the QDs in MC-SPT **(** Fig. S8). The results show that <1% of sAv-QD565 and sAv-QD605 are detected in the respective wrong spectral windows of 605/20 nm, and 565/40 nm, respectively. The results further show that ≈5% of both sAv-QD655 and sAv-QD705 are detected in the respective wrong spectral windows of >690 nm, and 655/20 nm, respectively. The level of spectral overlap of QD655 and QD705 can likely be decreased by the introduction of a fluorescence bandpass filter in the >690 nm channel as well as a narrower filter in the 655 channel, e.g. 655/10, however this would be done at the expense of both the fluorescence intensity and the localization precision of the QD655 and QD705.

### Application of Four Color MC-SPT with QDs

**Figure 6:**
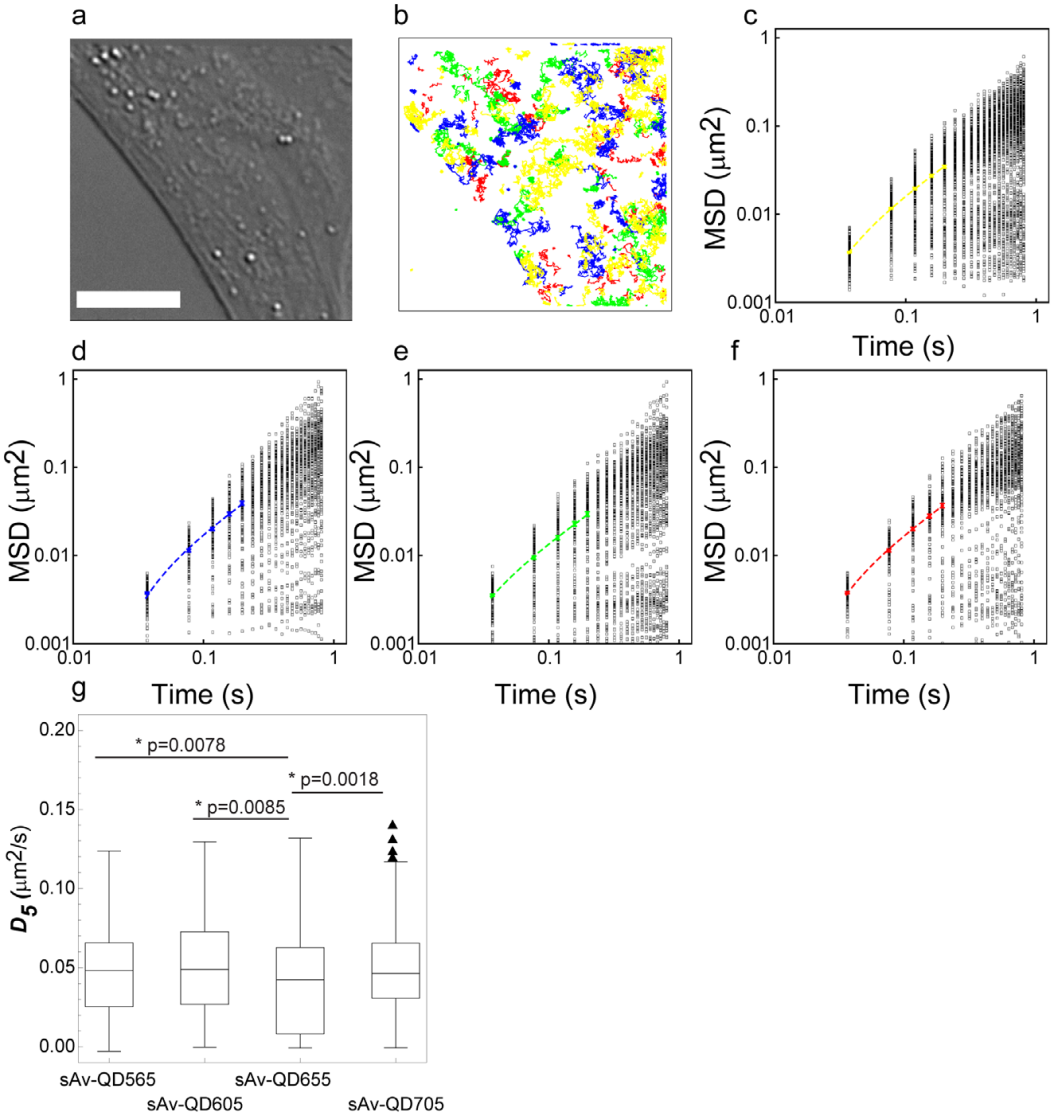
Four color MC-SPT of an artificially loaded lipid, biotin-cap-DPPE, in a live mouse embryo fibroblast (MEF). A synthetic lipid, biotin-cap-DPPE, was bulk loaded into the plasma membrane of live cells as described in the Methods section. Thus inserted lipids were subsequently labeled with a four color combination of sAv-QDs. (a) Differential interference contrast image of labeled cell. Scale bar equals 10 µm. (b) Superimposed single molecule trajectories for all trajectories, N, with, n>20 image frames from SPT analysis showing the cumulative lateral motion of lipids during a 1200 image frame acquisition (acquired at an image acquisition rate of ≈25 Hz for a time duration of ≈48 s) that were labeled with sAv-QD565 (yellow, N = 289), sAv-QD605 (blue, N = 142), sAv-QD655 (green, N = 162), and sAv-QD705 (red, N = 131). The distributions of the number of QDs that were detected in each image frame are shown in Fig. S10. The distributions of trajectory lengths for each image channel are shown in Fig. S11. (c-f) Log_10_ (MSD) versus log_10_(t) plots for all detected single molecule trajectories in (b) for the time intervals, t≤n t≤20 t, in each separate QD color channel where (c) sAv-QD565, (d) sAv-QD605, (e) sAv-QD655, and (f) sAv-QD705. The MSD curves for each single trajectory were curve fit at short time intervals, ∼40≤t≤∼200 ms, to determine the diffusion coefficient, D_5_, as described in the Methods section. The distributions of the fitted values of D_5_ for each trajectory are shown in (g) and in Fig. S12. A Kolmogorov-Smirnov statistical test of the differences in the population of the means of D_5_ for each sAv-QD type show that there is no statistical difference in the lateral dynamics of lipids that were labeled with either sAv-QD565, sAv-QD605, or sAv-QD705, but that there is a statistical difference between lipids that were labeled with sAv-QD655 as compared to the other sAv-QDs (p-values are shown in (g)). Also shown in (d-f) is the time averaged <MSD> at short time intervals, ∼40≤t≤∼200 ms, and the resulting linear fit to a free diffusion model. The resulting fit values for the time averaged <D_5_>(± a.s.e.) in this example are for c) sAv-QD565: 0.047±0.002 µm^2^/s, d) sAv-QD605: 0.052±0.003 µm^2^/s, e) sAv-QD655: 0.039±0.002 µm^2^/s, and f) sAv-QD705: 0.049±0.003 µm^2^/s.

To demonstrate the first application of parallel four color MC-SPT with QDs in live cells, we have imaged the lateral dynamics of artificially loaded lipids, biotin-cap-DPPE, in a live mouse embryo fibroblast (MEF). The artificially loaded lipids were labeled with a four color combination of sAv-QD565, sAv-QD605, sAv-QD655, and sAv-QD705, respectively and the lateral dynamics was imaged with 10****ms integration at an image acquisition rate of ≈25 Hz for 1200 image frames. These experiments were performed in the presence of 50 µM β-ME to suppress QD blinking and spectral shifting [21] [23]. A representative cell and superimposed particle trajectories are shown in Figures 6a–b. The MC-SPT analysis in this case resulted in a mean QD detection rate (±1 SD) per image frame of sAv-QD565: 42±3, sAv-QD605: 21±2, sAv-QD655: 28±2, sAv-QD705: 21±2, for a total detection rate of 112±5 QDs per image frame (Fig. S10). In the given example this corresponds to a QD labeling density over the area of the cell of sAv-QD565: 0.075±0.005 QDs/µm^2^, sAv-QD605: 0.037±0.004 QDs/µm^2^, sAv-QD655: 0.050±0.004 QDs/µm^2^, sAv-QD705: 0.037±0.004 QDs/µm^2^_,_ for an overall QD labeling density of 0.200±0.009 QDs/µm^2^ in all channels.

The resulting data analysis of all observed particle trajectories, N, longer than n >20 image frames, using classical MSD SPT analysis of the motion at short time intervals, 1≤n t≤5 corresponding to a time window of ≈40≤t≤≈200 ms is shown in Fig. 6c–g. These results show that there is a large amount of heterogeneity among the observed trajectories in the analyzed time window, ranging from a few immobile QDs to a vast majority of QDs that are mobile but over a very wide range of length and time scales (Fig. 6c–g, Fig. S12, and Table S6). The measured magnitudes of the diffusion rates, D_5_, are comparable to other similar measurements of the long range diffusion of lipids and membrane proteins in the plasma membrane of live cells, and which were acquired at similar acquisition rates (28).

A statistical analysis, the Kolmogorov-Smirnov statistical test, which does not assume a Gaussian distribution of the population, of the differences of the population means of the diffusion coefficient, D_5_, of the lateral dynamics of biotin-cap-DPPE that had been labeled with the various sAv-QDs show that there is a statistical difference between the lateral dynamics of the lipids that had been labeled with sAv-QD655 as compared to all other colors of QDs (sAv-QD565, sAv-QD605, and sAv-QD705). But there was no statistical difference in the lateral motion of lipids that had been labeled with either sAv-QD565, sAv-QD605, or sAv-QD705 (Fig. 6g and Table S7). The observed statistical significant difference in the lateral dynamics of lipids that were labeled with sAv-QD655 as opposed to all other sAv-QDs is a consequence of the detected larger proportion of low mobility lipids in this case. This is most likely a result of increased probe induced cross-linking with the sAv-QD655 as opposed to the other sAv-QDs. These results could in principal also be caused by steric hindrance due to the probe size although the FCS results in this case indicate that is not the case as sAv-QD705 are slightly larger than sAv-QD655.

### Conclusion

In order to optimize four color SPT, we have in this work performed a comprehensive single molecule imaging comparison of QD fluorescence intensities, and intensity fluctuations. We find that the ensemble average mean fluorescence intensity, I_QD_^on^, is strongly dependent on the QD peak emission, where the results ranked in order of decreasing I_QD_^on^ are I_QD625_^on^≈I_QD655_^ on^>I_QD605_^on^≈I_QD705_^on^>I_QD565_^on^≈I_QD585_^on^≈I_QD800_^on^>I_QD525_^on^.We have also shown that the relative fraction that QDs are in their bright emitting fluorescent “on” state, F_QD_^on^, is strongly dependent on the QD peak emission, with the major difference being that QD705s and QD800s, are primarily in a dark “off” state. The results from this analysis, ranked in order of decreasing F_QD_^on^are F_QD525_^on^≈F_QD565_^on^≈F_QD585_^on^≈F_QD605_^on^_≈_F_QD625_^on^_≈_F_QD655_^on^>>>F_QD705_^on^≈F_QD800_^on^ with an average fractional “on” time of 0.82±0.04 for QD525–QD655, and 0.21±0.08 for QD705 and QD800, respectively. In agreement with previous results [21], we also find that the fractional time that a QD is in its fluorescent bright “on” state is increased in the presence of small reducing agents, in this case 1 mM DTT, while the intensity of the fluorescent “on” state, within the precision and data analysis of the measurements, is approximately constant. The effect of addition of DTT is especially significant for near-IR QDs where the relative average fractional “on” time in the presence of 1 mM DTT is almost doubled. Finally, we have used FCS to measure the hydrodynamic size of various QD conjugates. In this case we show that the basic AMP-QD building block is equivalent in size to that of a mouse IgG1 while sAv-AMP-QDs are about twice that size (Fig. 5 and Table S3). We further show that because of the additional size that the water stabilizing surface coats add to the QD conjugates, there is only a minimal size gain advantage in working with more blue-shifted QDs in aqueous conditions.

Applying these results, we have demonstrated the experimental realization of four color MC-SPT by use of QD565, QD605, QD655, and QD705 to measure the lateral dynamics of a single type of an artificial lipid, biotin-cap-DPPE, in the plasma membrane of a live MEF with an image acquisition time of 10 ms and an image acquisition rate of ≈25 Hz. The use of four color MC-SPT in this case enables SPT measurements at much higher sample densities than is possible with a single probe. The MC-SPT results here show that there is a statistical significant difference in the population mean of the diffusion rate between lipids that are labeled with sAv-QD655 as opposed to lipids that are labeled with either sAv-QD565, sAv-QD605, or sAv-QD655 (Fig. 6g). This highlights the possible influence that the probe can have on the results in the case of SPT. Future applications of this initial demonstration of parallel four color MC-SPT will also extend to four color MC-SPT for the lateral dynamics of a combination of different lipids and proteins in a single live cell.

## Materials and Methods

### QD Sample Preparation for Imaging and FCS

The QDs used here were Qdot® ITK streptavidin (sAv) conjugates. These were purchased either in the form of Qdot® Strepavidin Conjugate Sampler Kits or individually from Invitrogen (Carlsbad, CA). The product specifications of these QDs are given in Table S1. These QDs are water stabilized with an amphiphillic poly (acrylic acid) layer [30] to which sAvhas been directly covalently conjugated. These QDs have been reported to have a range of ≈40–80 biotin binding sites. [25] All chemicals were reagent grade from Sigma-Aldrich (Brøndby, Denmark) except where specifically noted. For imaging and FCS experiments, we diluted QDs in 50 mM sodium borate, pH 8.5, containing 1% BSA (Sigma A-7906) at concentrations ranging from 1–10 nM immediately before use. For imaging, diluted QDs were non-specifically adsorbed to the surfaces of sample chambers, composed of 3″×1″ glass or fused silica slides, two strips of double adhesive tape and a #1½ glass coverslip, for a few minutes. Samples were subsequently extensively washed to remove non-adsorbed QDs with either 50 mM sodium borate, pH 8.5, containing 1% BSA with and without DTT. For FCS experiments, we used 8 well chamber slides (Lab-Tek^TM^II, VWR- Bie-Berntsen, Herlev, DK).

### Microscopy

All samples were imaged by use of wide-field illumination on an Olympus IX-81 inverted microscope (Olympus, Ballerup, Denmark) with a 150×1.45 NA UApo TIRFM Olympus oil immersion microscope objective and by use of a 100****W Hg arc lamp for fluorescence excitation. The excitation power at the objective back aperture plane and with a filter cube consisting of a HQ470/40 nm fluorescence excitation filter, a Q495LP dichroic mirror (Chroma Technology, Rockingham, VT) and a 500****LP emission filter was measured to be ≈35 mW/cm^2^. Data was acquired on an Andor DV887-ECS/BV EMCCD (Andor, Belfast, UK) and using Andor IQ software. The pixel size of this camera is 16×16 µm^2^. This combined set-up results in that the optical sampling is optimized according to the Nyquist criterion for light microscopy with a projected pixel size of ≈108 nm^2^. The quantum efficiency (QE) of this camera, according to the manufacturer, exceeds 90% from 500 nm to about 670 nm, dropping to about 85% at 700 nm and 70% at 800 nm (Fig. S2). Simultaneous multi-color single QD measurements were acquired using a QuadView image splitter (Bio-Science Aps, Gilleleje, Denmark) with dichromatic mirrors at Q585LP, Q630LP and Q690LP nm and with D565/40****m nm, D605/20****m nm, and D655/20****m nm fluorescence bandpass filters from Chroma Technologies (Rockingham, Vt, USA; Fig. S2).

### Image Analysis of QD Brightness and Fractional Intermittency

The position of single QDs in each image set was determined as has been described before. [28] In brief, an algorithm that automatically identified single QDs in an image sequence was constructed in Mathematica based on the following principles: QDs display discrete “on” and “off” fluorescence intensity states; a single QD exhibits only one “on” intensity state; and the magnitude of the “on” intensity state of a QD is constant over time and reproducible among all QDs. Having identified the positions of all single QDs, we generated frequency histograms of the fluorescence intensity as before by calculating the average pixel intensity over a 9×9 pixel array that was centered on the position of a specific single QD. These histograms were subsequently curve fit to the sum of a normal distribution, approximating the background intensity of the camera, P(I_off_), and an asymmetric normal distribution, multiplied by the instrument response function (IRF) of the microscope. The rationale for this fitting model is that the QD intermittency has an inverse power law dependence such that intermittency also frequently occurs at time scales much less that the integration rate of a measurement. This implies that several measurements are of QDs that are partly on and partly off [21], [22]. The fitting equation that we used and which describes the probability density function of the observed fluorescence intensities from a large number of single QDs during a finite observation time, t_obs_(5 or 10 ms in this case), is
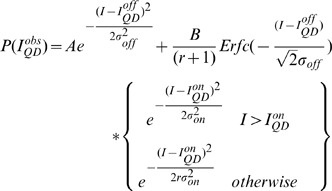
(1)where the probability density that the QD is its fluorescent dark “off” state is
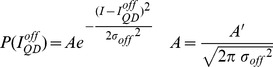
(2)and the probability density that the QD is in its fluorescent bright “on” state is



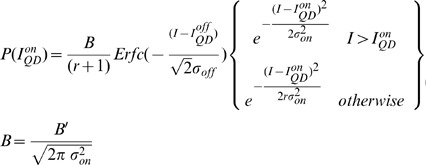
(3)In using these equations for curve fitting the data, the amplitudes, A and B, the mean fluorescence intensities, I_QD_^on/off^, the width of the mean fluorescence intensity distributions, σ^on/off^, and the asymmetry factor r were free parameters (Fig. 3). From the resulting fitted values, we can then calculate the mean fractional “on” time, F_QD_^on^, and the mean fractional “off” time, F_QD_^off^, which we define as
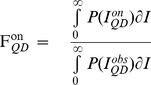
(4)

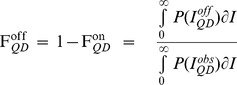
(5)


The resulting fit parameters for eight colors of QDs in the absence and presence of 1 mM DTT are given in Fig. 4 and Table S2.

### Fluorescence Correlation Spectroscopy (FCS)

The FCS measurements reported in this paper were made on a custom built multiphoton excitation microscope that has been described previously [31]. This setup is specially constructed on an Olympus IX70 microscope. The objective used in the experiments was a 60X, 1.2 NA water immersion objective. The excitation light source was a femtosecond Ti:Sa laser (Deep See, Spectra Physics, Mountain View, CA) and the excitation wavelength was 780 nm. The correlation data was collected at 50 kHz for ≈1 min and where each reported measurement is the average measurement from at least five independent measurements. The correlation data was curve fit to
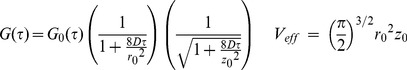
(6)where D is the diffusion coefficient, τ is the correlation time, and where r_0_ is beam waist in the radial direction and z_0_ is the beam waist in the axial direction, and V_eff_ is the excitation volume. In these experiments, we used Alexa488 labeled mouse IgG1 as a reference size standard with a known hydrodynamic radius of R_H_(Ms IgG1) = 5.6±0.2 nm [29] in order to calibrate the excitation volume, V_eff,_. The diffusion coefficient, D, is inversely dependent on the molecular size, R_H_, and is given by the Stokes-Einstein relation
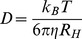
(7)where kB is the Boltzmann constant, T is the absolute temperature of the measurement, and η is the absolute viscosity of the solution. In this case, all FCS measurements were performed in 50 mM sodium borate pH 8.2 with 1% (w:v) BSA at RT (293 K). The relative size of molecular species when compared to a size standard, are independent of the viscosity and are given by



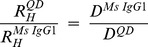
(8)In order to also convert the relative hydrodynamic radius values to absolute values, we estimated the viscosity, η, of the solution by assuming that BSA is a hard sphere in the sodium borate solution and by using
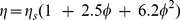
(9)where η_s_ is the viscosity of the solvent (here taken as that of pure water at 293****K (η_s_ = 1.002 cP)) and φ is the volume fraction occupied by BSA in the buffer. At 1% BSA (w:v) and using a R_H_ of 3.42 nm for BSA [32], the volume fraction, φ, is 0.015 yielding a viscosity, η, of 1.04 cP at 293****K.

### Quantification of Fluorescence Overlap of sAv-QD565, sAv-QD605, sAv-QD655, and sAv-QD705 in QuadView Configuration

In order to quantify the fluorescence overlap of the QDs in the QuadView microscope configuration, we acquired time-lapse image sequences of immobilized QDs of each color with 10 ms camera integration. Acquired image sequences were analyzed by a combination of ImageJ and Mathematica. To start, we used ImageJ to split the image sequences into quadrants of equal size, each corresponding to a specific spectral channel of 565/40 nm, 605/20 nm, 655/20 nm or >690 nm. Split image sequences were then concatenated into a single sequence and a maximum intensity projection image of the entire sequence was generated. We then used a Particle Tracker plug-in in ImageJ [33] to identify the positions of all QDs in the generated maximum intensity projection. The resulting QD positions along with the concatenated image sequence were subsequently imported into Mathematica in text format for further analysis. In Mathematica, we first calculated the mean pixel intensity, I_QD_, in ROIs of 7×7 pixels centered on each of the imported QD positions and in each image in the image sequence. We next counted all ROIs in each image, N_QD_^on^ (t), for each channel for which I_QD_/I_Bkgd_>1.01, where I_Bkgd_ is the mean pixel intensity of a similar sized ROI but which contained no QD. We next determined the fraction of the counted ROIs, N_QD_^on^ (t), in each spectral channel relative to the total sum of all ROIs in all four spectral channels, 
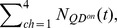
at each time point, t (Fig. S8).

### Localization Precision of MC-SPT with QDs

In order to determine the localization precision, we used the SPT analysis as described above to repeatedly determine the centroid position of single immobilized QDs. The centroids from several single QDs were then superimposed by subtraction of the mean centroid of each single QD. We next determined the spatial precision, δr, where we define δr = (δx^2^+δx^2^)^1/2^ where δx and δy are the standard deviations of the determined mean centroids in the x- and y- direction, respectively.

### Multicolor Single Particle Tracking (MC-SPT)

Simultaneous four color QD labeling was done by washing cells 3× in Dulbecco’s PBS with 0.1 g/L CaCl_2_ and 0.1 g/L MgCl_2_ (D-PBS) and by bulk loading cells with biotin-cap-DPPE (Avanti Polar Lipids) by use of fatty-acid free BSA (Sigma, A-8806) [34]. For this labeling, we prepared a 100 µg/ml lipid stock solution in absolute ethanol (stored at 4°C). Cells were then loaded by diluting the lipid stock to 1 µg/ml in 0.1% fatty-acid free BSA in D-PBS and by incubation for 5 minutes at RT. Lipid loaded cells were then washed three times in D-PBS and blocked for non-specific binding in D-PBS with 1% BSA for 1–2 minutes. Cells were then labeled with a four color combination of sAv-QDs with peak emissions at 565 nm (QD565), 605 nm (QD605), 655 nm (QD655), and 705 nm (QD705), respectively. For this labeling, we diluted purchased QDs stock solutions to 1 nM each in D-PBS with 1% BSA and filtered the solution with a 0.22 µm syringe filter. Cells were then labeled in 0.5 ml of the QD labeling solution for 2 minutes at RT after which binding was blocked by addition of 100 µl of 1 mM biotin and a further incubation at RT for 2 minutes. Labeled cells were finally washed 3X in D-PBS and imaged in D-PBS with 1% BSA and 50 µM β-mercaptoethanol (β-ME) (Sigma) to minimize QD blinking and spectral shifting [21]–[23]. Multi-color SPT on live cells was exclusively performed on the apical cell surface at room temperature.

### Single Particle Tracking Analysis

Single acquired time-lapse sequences were split into four quadrants, each corresponding to a single QD color, and analyzed independently by use of a Particle Tracker plug-in in ImageJ [33]. This analysis generates a text file containing the positions of the detected QD particle positions in each image frame as well as linked trajectories describing the motion of individual QDs in time. In this analysis, a major limitation to the use of QDs in SPT is apparent by the generation of a large number of short trajectories rather than a more desirable few very long continuous particle trajectories (Fig. S11). In order to minimize the number of inaccurate particle linking events, this analysis was done with very conservative particle linking criteria, corresponding to a particle link range of 5 image frames and a maximum allowed particle displacement of one pixel per image frame. In order to further analyze the detected particle motion, the thus generated data was post-processed in custom written Mathematica routines. This post-processing included further linking of particle trajectories by a coincidence search routine in time and space of all trajectories, with a minimal length greater than a cut-off value, here 20 frames, with other trajectories that coincided within space, δr, here set to 8 pixels, but not time. We next calculated the mean squared displacements (MSD) individually for each single trajectory, m, that contained n >20 image frames, and for time intervals, t < n t<20 t, where t = t_lag_ – (1/3) t_aq_ [7], [15]

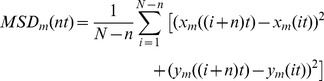
(10)where t_lag_ is the time interval between images, t_aq_ is the camera integration time, and N is the total number of frames in a trajectory. The MSD curves for each single trajectory, m, were curve fit at short time intervals, 1≤n t≤5 (corresponding to ∼40≤t≤∼200 ms) to a model for free diffusion with fit weights equal to the inverse variance (1/σ^2^)
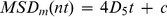
(11)where D5 is the diffusion coefficient and c is an off-set constant that has been proposed to be related to the spatial precision by which we can determine the position of a single molecule [7]. The mean diffusion coefficient of all trajectories for a given molecule and label was also determined by calculating the average mean squared displacements <MSD(nt)> at each time interval, n t, for each single trajectory, m



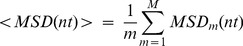
(12)The average diffusion coefficient was then determined again by curve fitting the initial five time points, with fit weights equal to the inverse variance (1/σ^2^), to
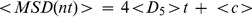
(13)where <D_5_> is the mean diffusion coefficient for a given molecule and label

### Statistical Analysis

The non-parametric Kolmogorov-Smirnov test (K-S test) was used to evaluate the differences between the different populations of the determined diffusion coefficient *D_5_*. The K-S test quantifies the distance between the cumulative density function of the two test populations. The null hypothesis is that the two test populations are drawn from the same distribution. The distributions are considered continuous, but are otherwise not restricted.

## Supporting Information

Figure S1 **Cumulative frequency histograms of fluorescence intensities of single sAv-QD525, sAv-QD585, sAv-QD625, and AMP-QD800. Cumulative frequency histograms of the background subtracted fluorescence intensities per pixel of 9×9 pixel arrays of identified single QDs in absence of DTT (left), and in presence of 1 mM DTT (right).** These histograms were generated from 3 fields of view in total containing between ∼100≤n≤∼600 single QDs that were each imaged for m = 300 image frames. The total sampling points for each histogram (m n) were ∼30,000≤m n≤∼180,000. The mean fluorescence intensities, I_QD_^on^, and fractional intermittency times, F_QD_^on^, were determined by non-linear curve fitting (red dashed line) as described in the Methods section.
(TIF)

Figure S2 **QuadView microscope filter configuration. Parallel multi-color single QD measurements were acquired using a QuadView image splitter (Bio-Science Aps, Gilleleje, Denmark) with dichromatic mirrors Q585LP, Q630LP and Q690LP and with D565/40 m, D605/20 m, and D655/20 m fluorescence bandpass filters from Chroma Technologies (Rockingham, Vt, USA).** All imaging was done on an Andor DV887-ECS/BV EMCCD (Belfast, Northern Ireland). The quantum efficiency (QE, dashed line) of this camera according to the technical specifications of the manufacturer is approximately independent of the wavelength over a spectral range of ∼500–∼670 nm, with an approximate decrease of 5% at 700 nm, and about 25% at 800 nm. The emission spectra of sAv-QD565 (orange line), QD605 (red line), QD655 (dark red line), and QD705 (black line) were acquired on a NanoDrop 3300 Fluorospectrometer (Saveen & Werner, Limhamn, Sweden).
(TIF)

Figure S3 **Representative fluorescence intensity time traces of single sAv-QD525, sAv-QD585, sAv-QD625, and AMP-QD800.** Representative, background subtracted, integrated fluorescence intensities, as a function of time, of identified single QDs that had been absorbed non-specifically to a glass coverslip in the absence of DTT (left) and in the presence of 1 mM DTT (right). Images of QDs with different peak emissions were acquired under identical conditions with continuous blue illumination with 5 ms integration at a frame rate of ∼25 Hz for 300 image frames. Also shown are the fluorescence intensity fluctuations of the background (grey lines).
(TIF)

Figure S4 **Localization precision of single sAv-QD525, sAv-QD565, sAv-QD585, sAv-QD605, sAv-QD625, sAv-QD655, QD705, and AMP-QD800:** The localization precision of single QD imaging with 5 ms camera integration times was determined by time-lapse imaging and SPT analysis of QDs that had been immobilized on glass. Shown are the determined centroids from N independent single QDs (black points) and the mean centroid (±1 SD; red) of all centroids, where superpositioning in the center of a projected pixel was done by first subtracting the mean centroid of each single QD, respectively. The centroids are displayed on the projected pixel array of the EMCCD (Projected pixel size of ≈108 nm). The localization precision, δr, was found to be (a) sAv-QD525: δr≈30 nm (N = 254), (b) sAv-QD565: δr≈22 nm (N = 1441), (c) sAv-QD585: δr≈22 nm (N = 1187), (d) sAv-QD605: δr≈14 nm (N = 8598), (e) sAv-QD625: δr≈16 nm (N = 1155), (f) sAv-QD655: δr≈14 nm (N = 2979), (g) sAv-QD705: δr≈16 nm (N = 427), and (h) AMP-QD800: δr≈23 nm (N = 337). Scale bar is equal to 100 nm.
(TIF)

Figure S5 **Localization precision of single sAv-QD525, sAv-QD565, sAv-QD585, sAv-QD605, sAv-QD625, sAv-QD655, sAv-QD705, and AMP-QD800 as a function of the mean fluorescence intensity, I_QD_^on^.** The localization precision of single QD imaging at 5 ms image integration was determined by time-lapse imaging and SPT analysis of QDs that had been immobilized on glass. This plot indicates that the maximum achievable localization precision, δr = (δx^2^+δy^2^)^1/2^, of the described microscope configuration and analysis methodology is δx = δy≈10 nm. This corresponds to a maximum localization precision of ≈1/10 the projected pixel size along either the x- or y-axis. The thus determined precision provides an estimate of the minimum localization precision in the case of completely stationary QDs. However, the precision will always be greater for non-stationary QDs as a result of mobility during the image integration time, t_Aq_. In this case, the precision will also depend on t_Aq_, the particle diffusion rate, D, and the mode of diffusion, i.e. for normal diffusion the precision would be δr = (δx^2^+δx^2^+4 D t_Aq_)^1/2^. It is noteworthy that the precision of SPT measurements is significantly improved in the presence of nanodomains.
(TIF)

Figure S6 **QD fluorescence brightness in QuadView microscope configuration.** Representative single image frame comparison of the fluorescence intensity of QD565, QD605, QD655, and QD705 in the QuadView microscope configuration. Time lapse images in each instance were acquired with 10 ms camera integration using a Hg arc lamp, a 470/40 nm bandpass excitation filter, a 510LP emission filter, a 150X, 1.45 NA objective, a QuadView image splitter and an Andor EMCCD BV887. All images are displayed at identical brightness and contrast ratios and are hence directly comparable.
(TIF)

Figure S7 **Cumulative frequency histograms of fluorescence intensities of single sAv-QD565, sAv-QD605, sAv-QD655, and sAv-QD705 in QuadView configuration.** Cumulative frequency histograms of the background subtracted fluorescence intensities per pixel of 9×9 pixel arrays of identified single QDs in absence of DTT. These histograms were generated from one field of view in total containing between ∼60≤n≤∼270 single QDs that were each imaged for m = 300 image frames. The total sampling points for each histogram (m n) were ∼18,000≤m n≤∼81,000. Ensemble average mean fluorescence intensities, I_QD_^on^, and fractional intermittency times, F_QD_^on^, were determined by non-linear curve fitting (red dashed line) as described in the Methods section.
(TIF)

Figure S8
**Quantification of fluorescence overlap of sAv-QD565, sAv-QD605, sAv-QD655, and sAv-QD705 in QuadView configuration.** The fraction of the number of detected fluorescent QDs, N_QD_^on^(t), in each respective color channel at each time point, t, relative to the total number of detected QDs, n all four spectral channels, 
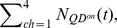
 was determined as described in the Methods section. The results show that <1% of sAv-QD565 are also detected in the 605/20 nm spectral window but none are detected in either the 655/20 nm or the >690 nm spectral windows (top left). Similarly, <1% of sAv-QD605 are also detected in the 565/40 nm spectral window but none are detected in either the 655/20 nm or the >690 nm spectral windows (top right). Furthermore, ≈5% of sAv-QD655 are detected in the upper spectral window of >690 nm but none are detected in ether the 605/20 nm or the 565/40 nm spectral windows. Finally, ≈5% of sAv-QD705 are also detected in the 655/20 nm spectral window but none are detected in ether the 605/20 nm or the 565/40 nm spectral windows.(TIF)

Figure S9 **Localization precision of single QD565, QD605, QD655, and QD705 in the QuadView configuration.** The localization precision of MC-SPT ín the QuadView configuration with 10 ms image integration was determined by time-lapse imaging and SPT analysis of QDs that had been immobilized on glass. Shown are the determined centroids of N single independent QDs (black points) and the mean centroid (±1 standard deviation; red) of all superimposed centroids, where superpositioning in the center of a projected pixel was done by first subtracting the mean centroid of each single QD respectively. The centroids are displayed on the projected pixel array of the EMCCD (Projected pixel size of ≈108 nm). The localization precision, δr, of the MC-SPT was found to be (a) sAv-QD565: δr≈21 nm (N = 1028), (b) sAv-QD605: δr≈14 nm (N = 1637), (c) sAv-QD655: δr≈15 nm (N = 2689), and (d) sAv-QD705: δr≈28 nm (N = 514). Scale bar is equal to 100 nm.
(TIF)

Figure S10 **Sample distributions of the number of detected QDs in each image frame in each image channel for the MC-SPT example in** **Fig. 6** **.** The mean ±1 SD of the number of detected sAv-QDs in each image channel for the example in Fig. 6 using the SPT analysis as described in the Methods section was for a) sAv-QD565: 42±3, b) sAv-QD605: 21±2, c) sAv-QD655: 28±2, and d) sAv-QD705: 21±2.The total mean ±1 standard deviation of the detected sAv-QDs for all channels were 112±5. In the given example this corresponds to a single QD labeling density of a) sAv-QD565: 0.075±0.005 QDs/µm^2^, b) sAv-QD605: 0.037±0.004 QDs/µm^2^, c) sAv-QD655: 0.050±0.004 QDs/µm^2^, and d) sAv-QD705: 0.037±0.004 QDs/µm^2^. The overall labeling density for all QDs in all channels was 0.200±0.009 QDs/µm^2^.
(TIF)

Figure S11 **Sample distributions of the length of the detected QD trajectories in each image channel for the MC-SPT example in** **Fig. 6** **.** A result of the QD blinking is that SPT with QDs results in many short trajectories such that the number of trajectories is much greater than the number of detected QDs in each image frame. In the given example in Fig. 6, SPT analysis resulted in n trajectories with a length of mean ± SEM. steps of a) sAv-QD565: 9±0.1 (n = 4,356), b) sAv-QD605: 15±0.4 (n = 1,367), c) sAv-QD655: 22±0.6 (n = 1,398), and d) sAv-QD705: 7±0.1 (n = 2,718) steps. Of these trajectories we analyzed only those that were longer than 20 steps. The corresponding numbers for these trajectories were for a) sAv-QD565: 83±6 (n = 289), b) sAv-QD605: 121±16 (n = 142), c) sAv-QD655: 161±20 (n = 162), and d) sAv-QD705: 65±11 (n = 131). The length of the entire image sequence was 1200 image frame acquired at an image acquisition rate of ≈25 Hz corresponding to a total duration of ≈48.3 s.
(TIF)

Figure S12 **Fitted diffusion coefficients, D_5_, for biotin-cap-DPPE for each sAv-QD conjugate in each image channel for the MC-SPT example in** **Figure 6** **.** The diffusion coefficient, D_5_, was determined by calculating the MSD curve for each trajectory that were longer than 20 steps, and by curve fitting the initial five points, t < n t <5 t, of each trajectory, as is described in the Methods section. The entire distribution of the fitted values of D_5_ are shown for all trajectories that were labeled with a) sAv-QD565 (N = 289), b) sAv-QD605 (N = 142), c) sAv-QD655 (N = 162), and d) sAv-QD705 (N = 131). The mean diffusion coefficient, <D_5_>, (± SEM) was for a) sAv-QD565: 0.047±0.002 µm^2^/s, b) sAv-QD605: 0.052±0.003 µm^2^/s, c) sAv-QD655: 0.039±0.002 µm^2^/s, and d) sAv-QD705: 0.049±0.003 µm^2^/s.
(TIF)

Table S1 **Product specifications of investigated QDs.** The reported molar extinction coefficients are those given by the manufacturer.
(DOC)

Table S2 **Quantification of mean intensity, I_QD_^on^, and fractional intermittency times, F_QD_^on^, of single QDs with 500 LP Emission Filter Microscope Configuration.**
(DOC)

Table S3 **Hydrodynamic Radii of QDs.**
(DOC)

Table S4 **Quantification of fluorescence intensities, I_QD_^on^, and fractional intermittency times, F_QD_^on^, of single QDs with QuadView microscope filter configuration.**
(DOC)

Table S5 **Localization precision of single QD imaging.**
(DOC)

Table S6 **Summary of MC-SPT results for four color tracking of biotin-cap-DPPE labeled with each type of sAv-QD.**
(DOC)

Table S7 **p-values for non-parametric two-sided Kolmogorov-Smirnov test of the diffusion coefficient of biotin-cap-DPPE in live MEFs.** White shade = Statistical significant difference at confidence level of α ≤ 0.05. Gray shade = No statistical significant difference at confidence level of α ≤ 0.05.
(DOC)

Movie S1 **QD fluorescence intensity.** Representative video comparison of the fluorescence intensities of QD525, QD565, QD585, QD605, QD625, QD655, QD705, and QD800. Time lapse images in each instance were acquired with 5 ms camera integration at an image acquisition rate of ≈25 Hz using a Hg arc lamp, a 470/40 nm bandpass excitation filter, a 500****LP emission filter, a 150X, 1.45 NA objective, and an Andor EMCCD BV887. All images are displayed at identical brightness and contrast ratios and are hence directly comparable. Scale bar equals 10 µm.
(AVI)
