# Supplementary figures and images for: Multi-Color Single Particle Tracking with Quantum Dots
Source: PLoS One. 2012 Nov 14;7(11):e48521. doi: 10.1371/journal.pone.0048521 (PMC3498293; doi:10.1371/journal.pone.0048521)

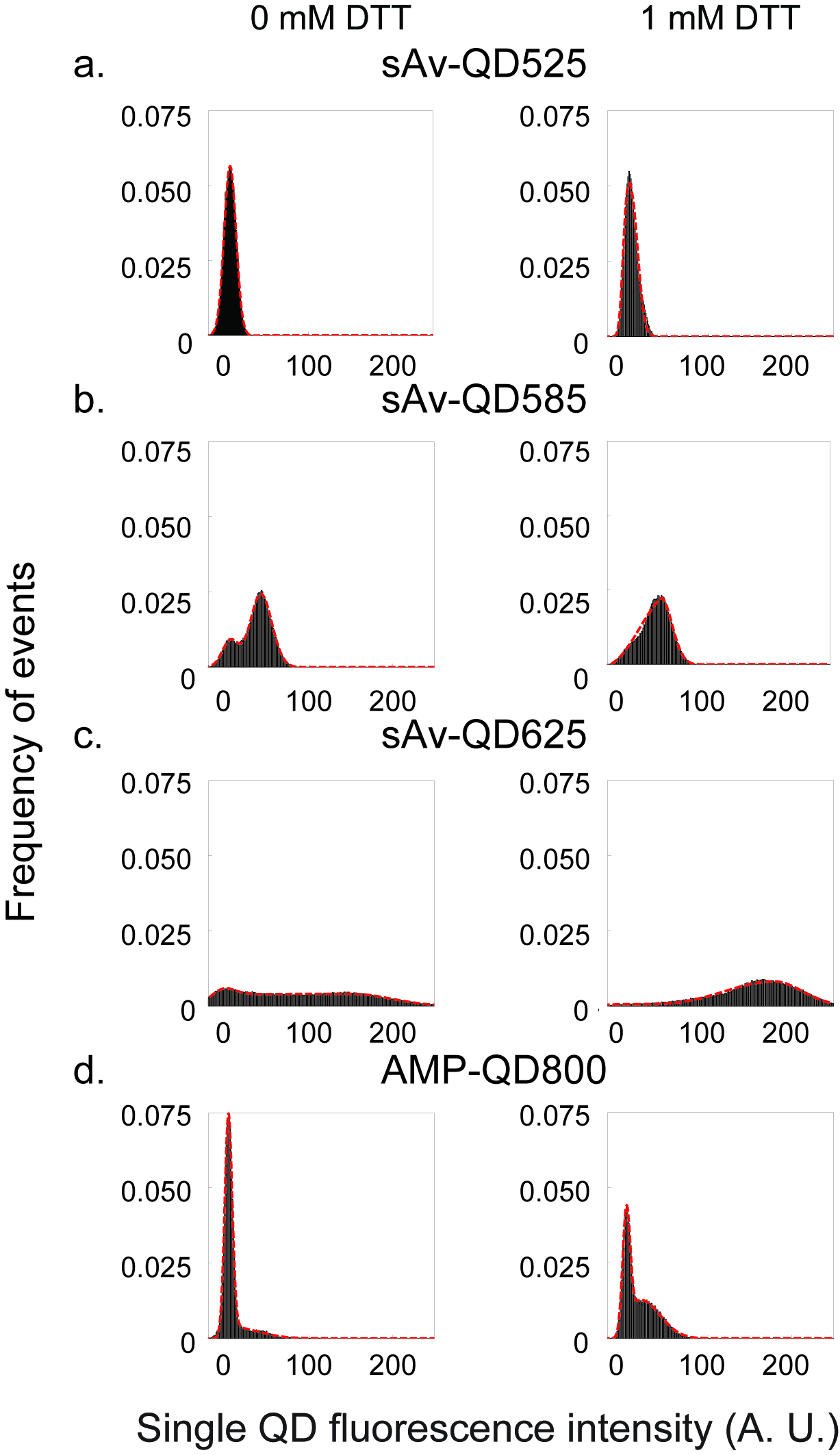

Supplement: Figure S1 — Cumulative frequency histograms of fluorescence intensities of single sAv-QD525, sAv-QD585, sAv-QD625, and AMP-QD800. Cumulative frequency histograms of the background subtracted fluorescence intensities per pixel of 9×9 pixel arrays of identified single QDs in absence of DTT (left), and in presence of 1 mM DTT (right). These histograms were generated from 3 fields of view in total containing between ∼100≤n≤∼600 single QDs that were each imaged for m = 300 image frames. The total sampling points for each histogram (m n) were ∼30,000≤m n≤∼180,000. The mean fluorescence intensities, IQD on, and fractional intermittency times, FQD on, were determined by non-linear curve fitting (red dashed line) as described in the Methods section. (TIF) [file pone.0048521.s001.tif]

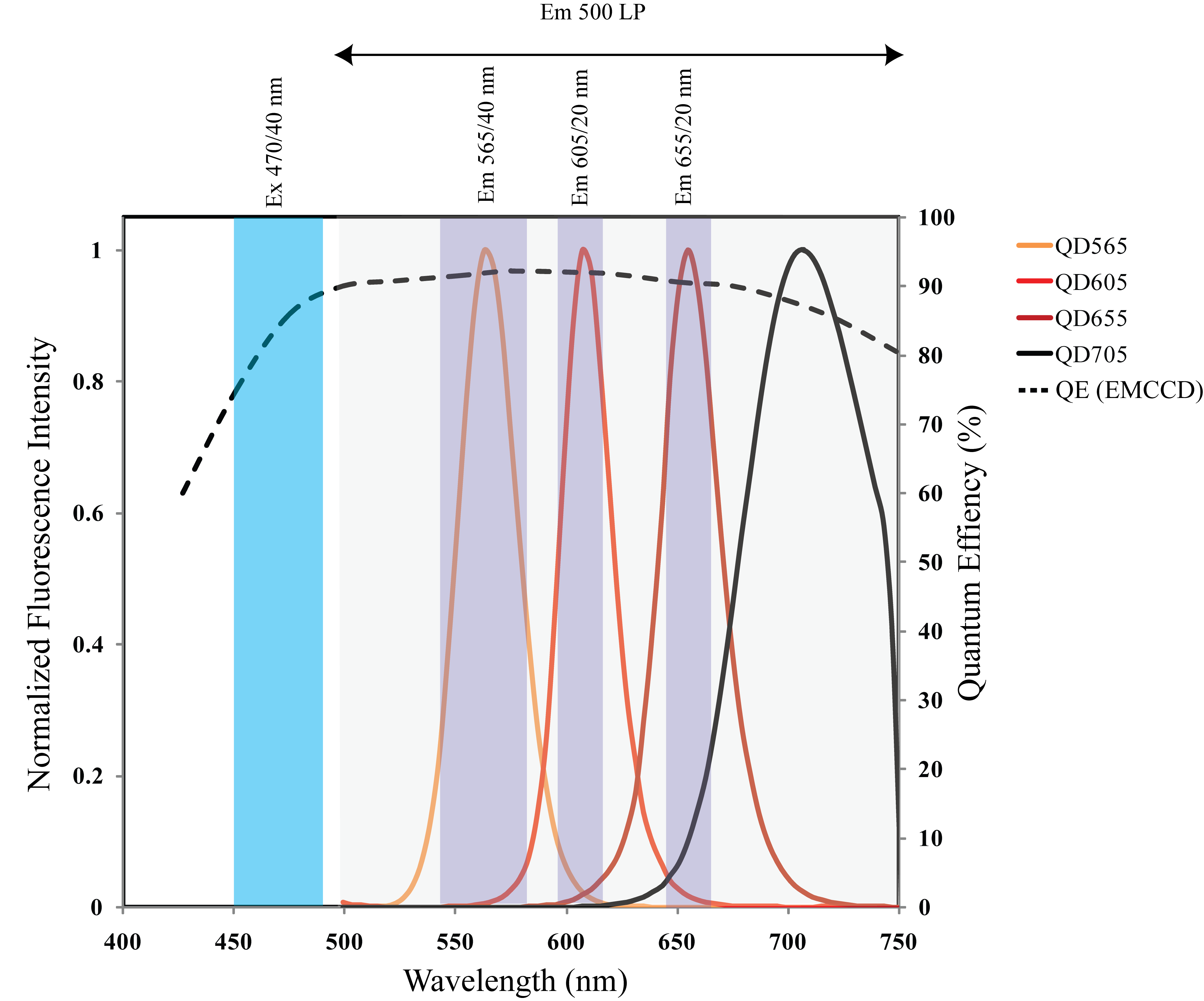

Supplement: Figure S2 — QuadView microscope filter configuration. Parallel multi-color single QD measurements were acquired using a QuadView image splitter (Bio-Science Aps, Gilleleje, Denmark) with dichromatic mirrors Q585LP, Q630LP and Q690LP and with D565/40 m, D605/20 m, and D655/20 m fluorescence bandpass filters from Chroma Technologies (Rockingham, Vt, USA). All imaging was done on an Andor DV887-ECS/BV EMCCD (Belfast, Northern Ireland). The quantum efficiency (QE, dashed line) of this camera according to the technical specifications of the manufacturer is approximately independent of the wavelength over a spectral range of ∼500–∼670 nm, with an approximate decrease of 5% at 700 nm, and about 25% at 800 nm. The emission spectra of sAv-QD565 (orange line), QD605 (red line), QD655 (dark red line), and QD705 (black line) were acquired on a NanoDrop 3300 Fluorospectrometer (Saveen & Werner, Limhamn, Sweden). (TIF) [file pone.0048521.s002.tif]

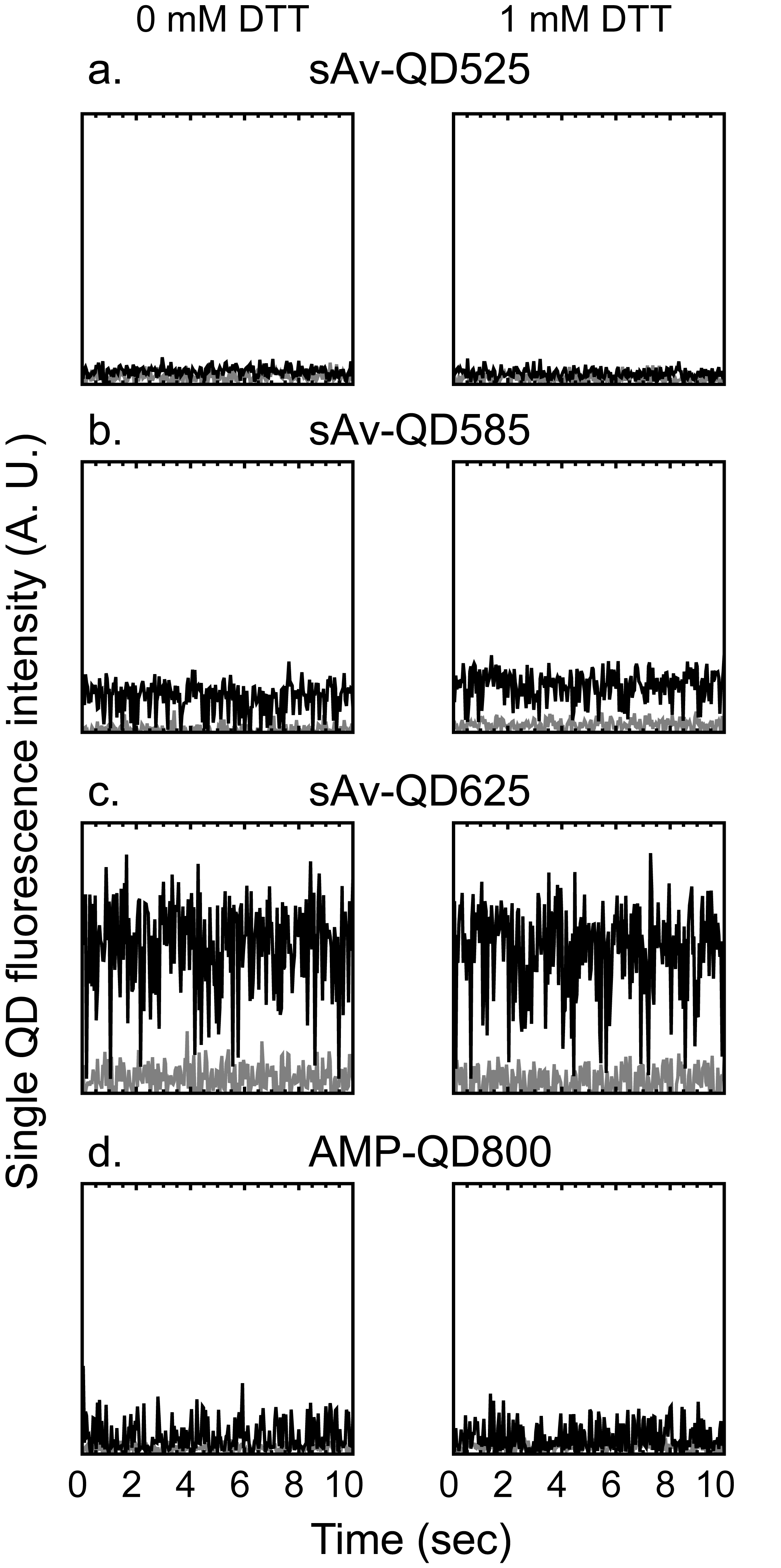

Supplement: Figure S3 — Representative fluorescence intensity time traces of single sAv-QD525, sAv-QD585, sAv-QD625, and AMP-QD800. Representative, background subtracted, integrated fluorescence intensities, as a function of time, of identified single QDs that had been absorbed non-specifically to a glass coverslip in the absence of DTT (left) and in the presence of 1 mM DTT (right). Images of QDs with different peak emissions were acquired under identical conditions with continuous blue illumination with 5 ms integration at a frame rate of ∼25 Hz for 300 image frames. Also shown are the fluorescence intensity fluctuations of the background (grey lines). (TIF) [file pone.0048521.s003.tif]

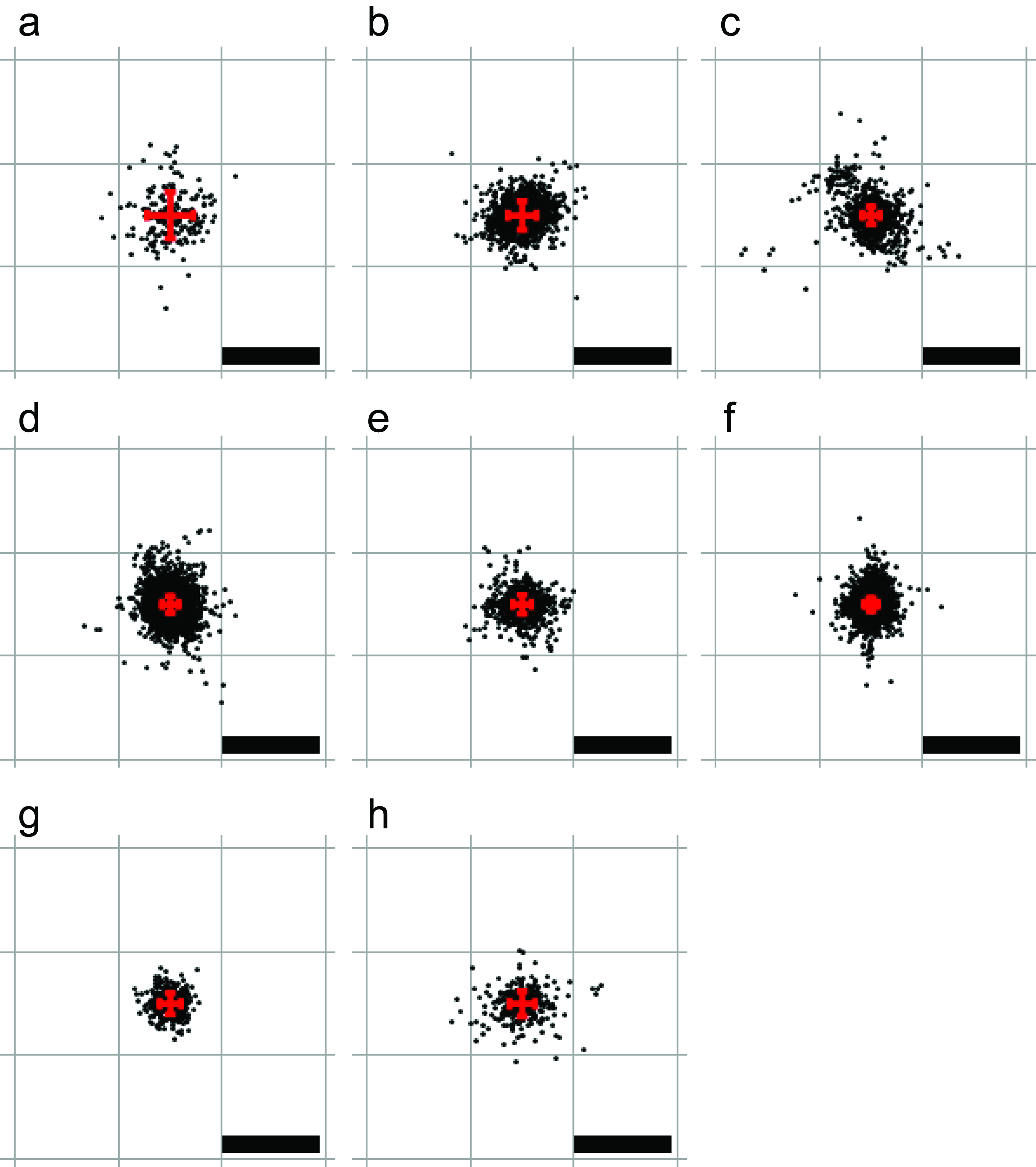

Supplement: Figure S4 — Localization precision of single sAv-QD525, sAv-QD565, sAv-QD585, sAv-QD605, sAv-QD625, sAv-QD655, QD705, and AMP-QD800: The localization precision of single QD imaging with 5 ms camera integration times was determined by time-lapse imaging and SPT analysis of QDs that had been immobilized on glass. Shown are the determined centroids from N independent single QDs (black points) and the mean centroid (±1 SD; red) of all centroids, where superpositioning in the center of a projected pixel was done by first subtracting the mean centroid of each single QD, respectively. The centroids are displayed on the projected pixel array of the EMCCD (Projected pixel size of ≈108 nm). The localization precision, δr, was found to be (a) sAv-QD525: δr≈30 nm (N = 254), (b) sAv-QD565: δr≈22 nm (N = 1441), (c) sAv-QD585: δr≈22 nm (N = 1187), (d) sAv-QD605: δr≈14 nm (N = 8598), (e) sAv-QD625: δr≈16 nm (N = 1155), (f) sAv-QD655: δr≈14 nm (N = 2979), (g) sAv-QD705: δr≈16 nm (N = 427), and (h) AMP-QD800: δr≈23 nm (N = 337). Scale bar is equal to 100 nm. (TIF) [file pone.0048521.s004.tif]

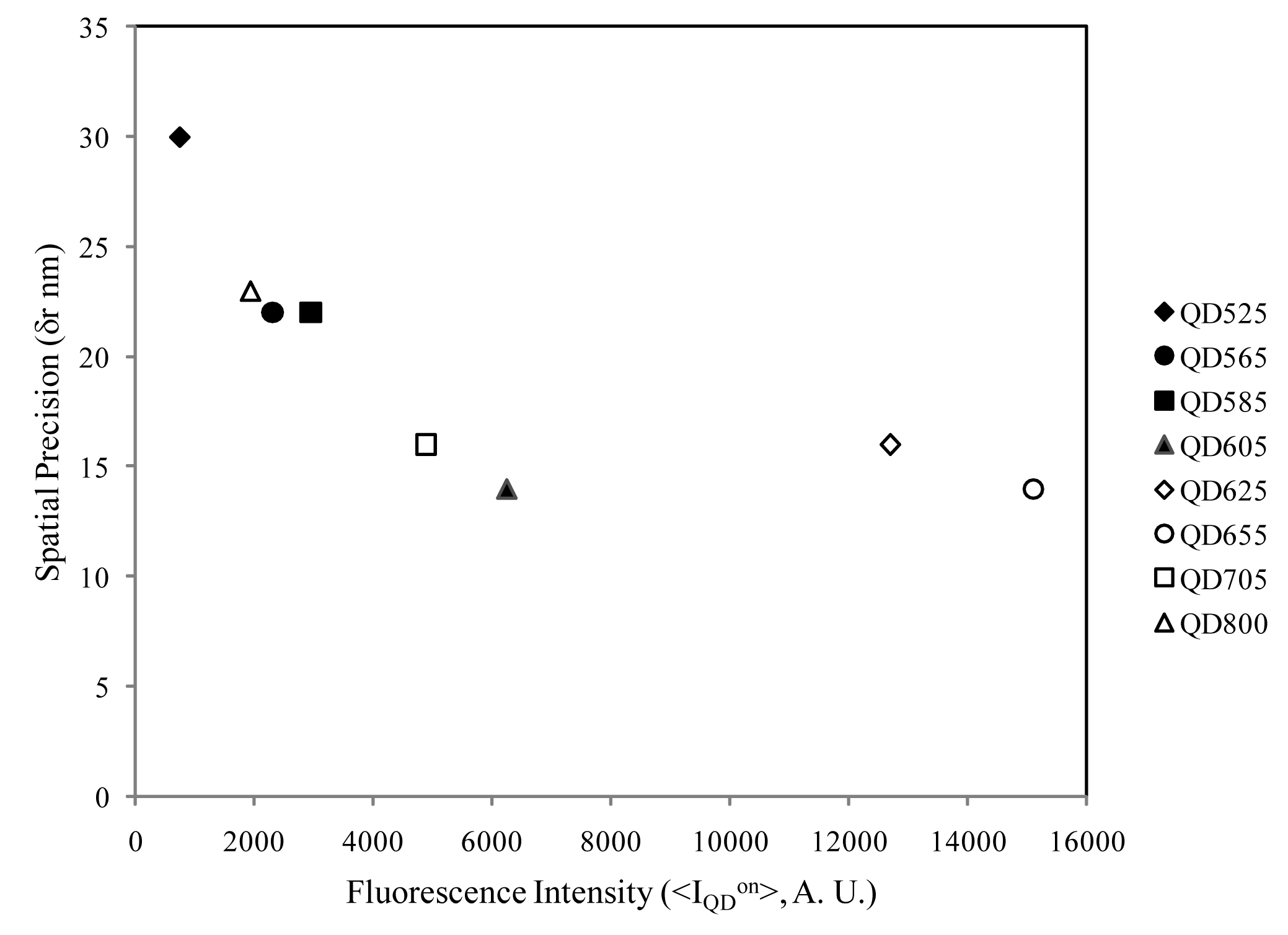

Supplement: Figure S5 — Localization precision of single sAv-QD525, sAv-QD565, sAv-QD585, sAv-QD605, sAv-QD625, sAv-QD655, sAv-QD705, and AMP-QD800 as a function of the mean fluorescence intensity, IQDon. The localization precision of single QD imaging at 5 ms image integration was determined by time-lapse imaging and SPT analysis of QDs that had been immobilized on glass. This plot indicates that the maximum achievable localization precision, δr = (δx2+δy2)1/2, of the described microscope configuration and analysis methodology is δx = δy≈10 nm. This corresponds to a maximum localization precision of ≈1/10 the projected pixel size along either the x- or y-axis. The thus determined precision provides an estimate of the minimum localization precision in the case of completely stationary QDs. However, the precision will always be greater for non-stationary QDs as a result of mobility during the image integration time, tAq. In this case, the precision will also depend on tAq, the particle diffusion rate, D, and the mode of diffusion, i.e. for normal diffusion the precision would be δr = (δx2+δx2+4 D tAq)1/2. It is noteworthy that the precision of SPT measurements is significantly improved in the presence of nanodomains. (TIF) [file pone.0048521.s005.tif]

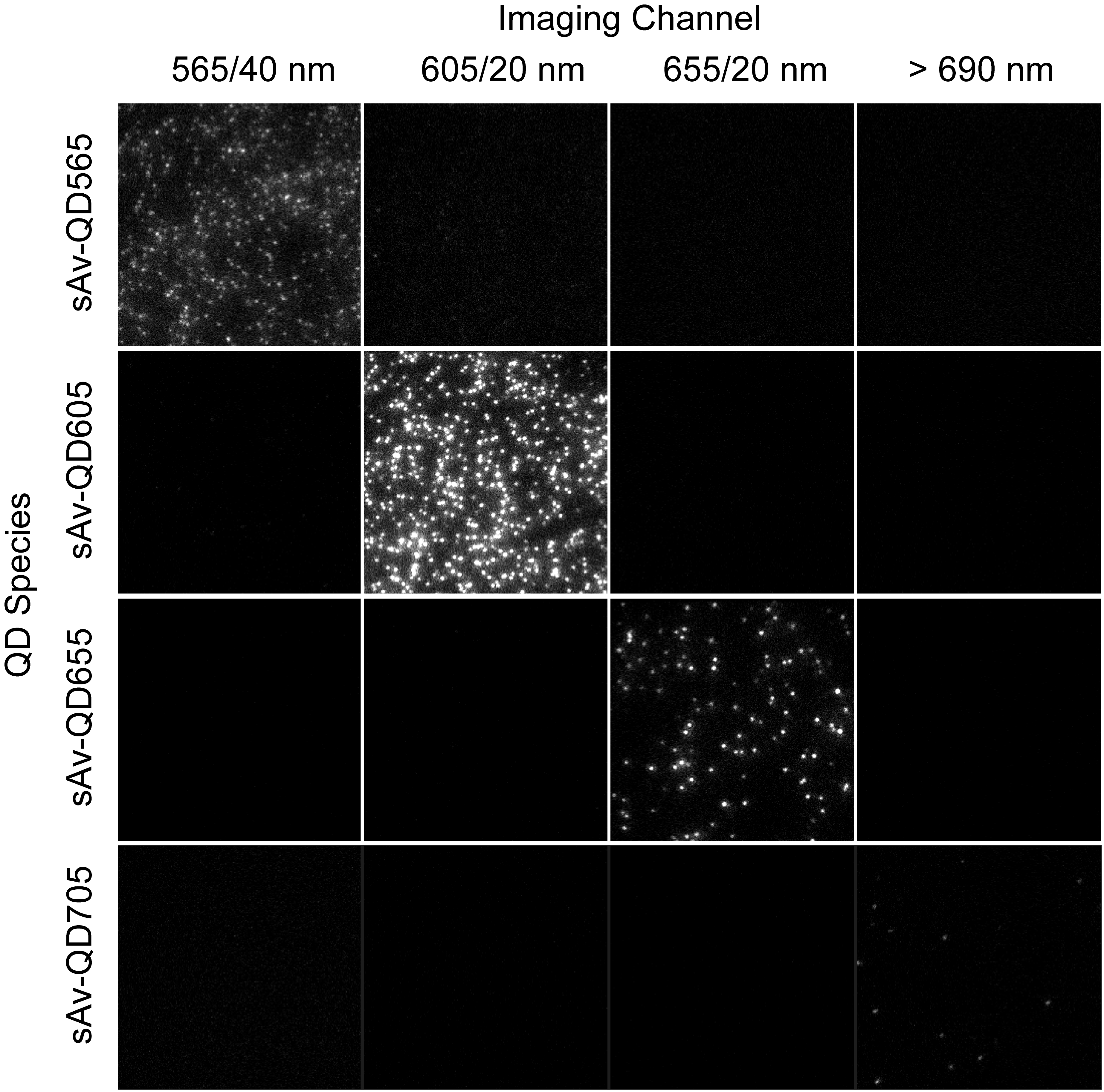

Supplement: Figure S6 — QD fluorescence brightness in QuadView microscope configuration. Representative single image frame comparison of the fluorescence intensity of QD565, QD605, QD655, and QD705 in the QuadView microscope configuration. Time lapse images in each instance were acquired with 10 ms camera integration using a Hg arc lamp, a 470/40 nm bandpass excitation filter, a 510LP emission filter, a 150X, 1.45 NA objective, a QuadView image splitter and an Andor EMCCD BV887. All images are displayed at identical brightness and contrast ratios and are hence directly comparable. (TIF) [file pone.0048521.s006.tif]

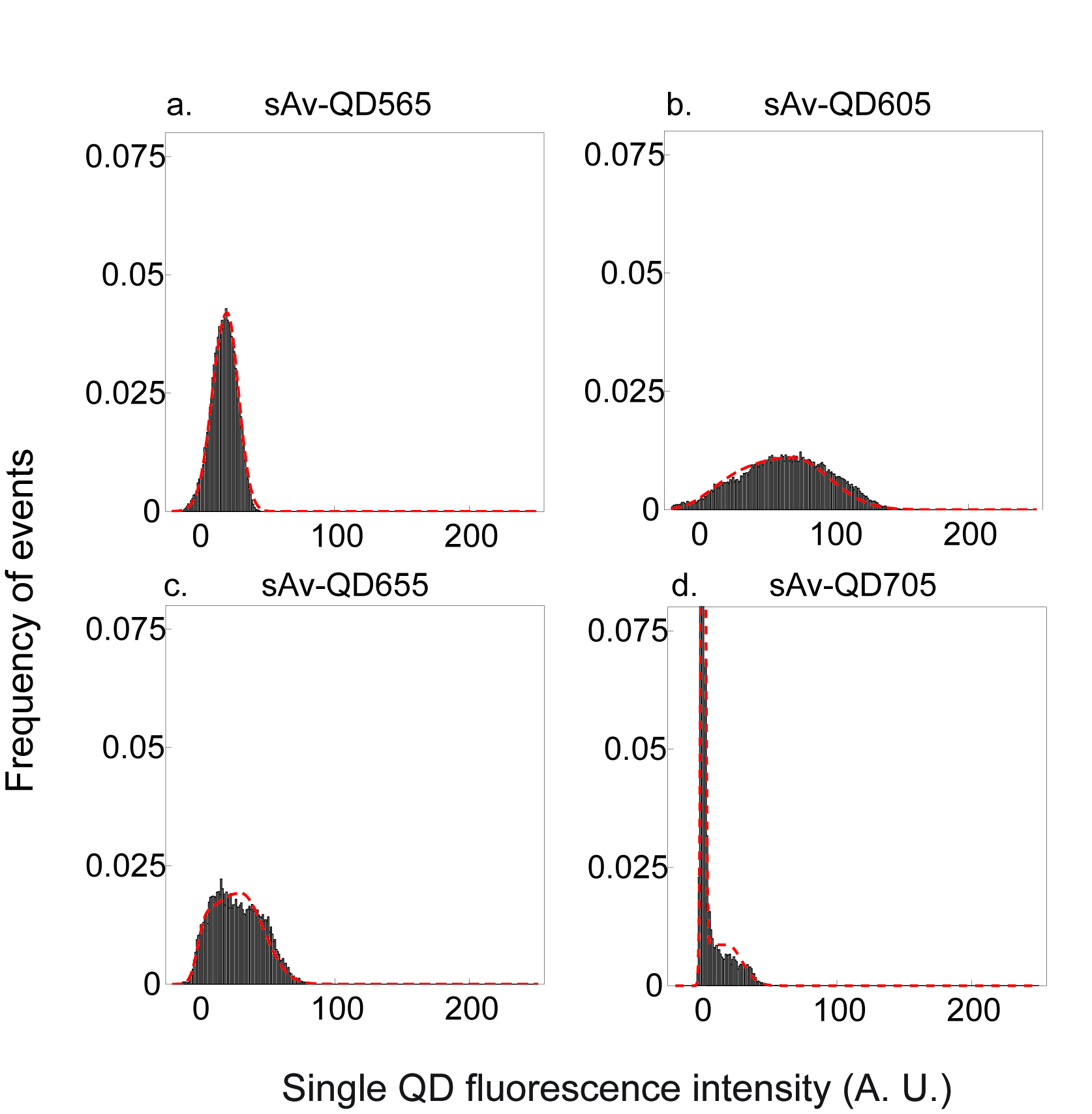

Supplement: Figure S7 — Cumulative frequency histograms of fluorescence intensities of single sAv-QD565, sAv-QD605, sAv-QD655, and sAv-QD705 in QuadView configuration. Cumulative frequency histograms of the background subtracted fluorescence intensities per pixel of 9×9 pixel arrays of identified single QDs in absence of DTT. These histograms were generated from one field of view in total containing between ∼60≤n≤∼270 single QDs that were each imaged for m = 300 image frames. The total sampling points for each histogram (m n) were ∼18,000≤m n≤∼81,000. Ensemble average mean fluorescence intensities, IQD on, and fractional intermittency times, FQD on, were determined by non-linear curve fitting (red dashed line) as described in the Methods section. (TIF) [file pone.0048521.s007.tif]

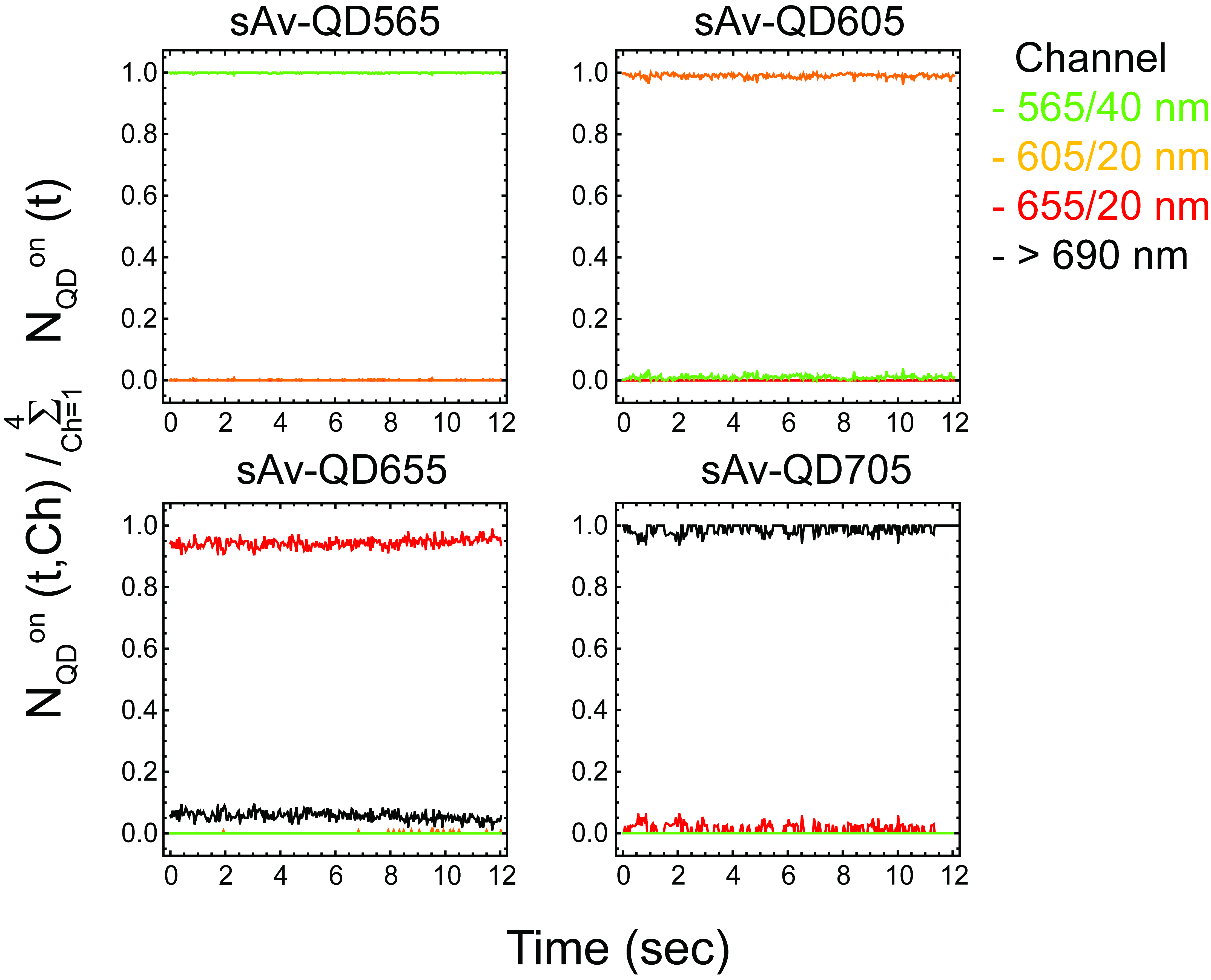

Supplement: Figure S8 — Quantification of fluorescence overlap of sAv-QD565, sAv-QD605, sAv-QD655, and sAv-QD705 in QuadView configuration. The fraction of the number of detected fluorescent QDs, NQD on(t), in each respective color channel at each time point, t, relative to the total number of detected QDs, n all four spectral channels, was determined as described in the Methods section. The results show that <1% of sAv-QD565 are also detected in the 605/20 nm spectral window but none are detected in either the 655/20 nm or the >690 nm spectral windows (top left). Similarly, <1% of sAv-QD605 are also detected in the 565/40 nm spectral window but none are detected in either the 655/20 nm or the >690 nm spectral windows (top right). Furthermore, ≈5% of sAv-QD655 are detected in the upper spectral window of >690 nm but none are detected in ether the 605/20 nm or the 565/40 nm spectral windows. Finally, ≈5% of sAv-QD705 are also detected in the 655/20 nm spectral window but none are detected in ether the 605/20 nm or the 565/40 nm spectral windows. (TIF) [file pone.0048521.s008.tif]

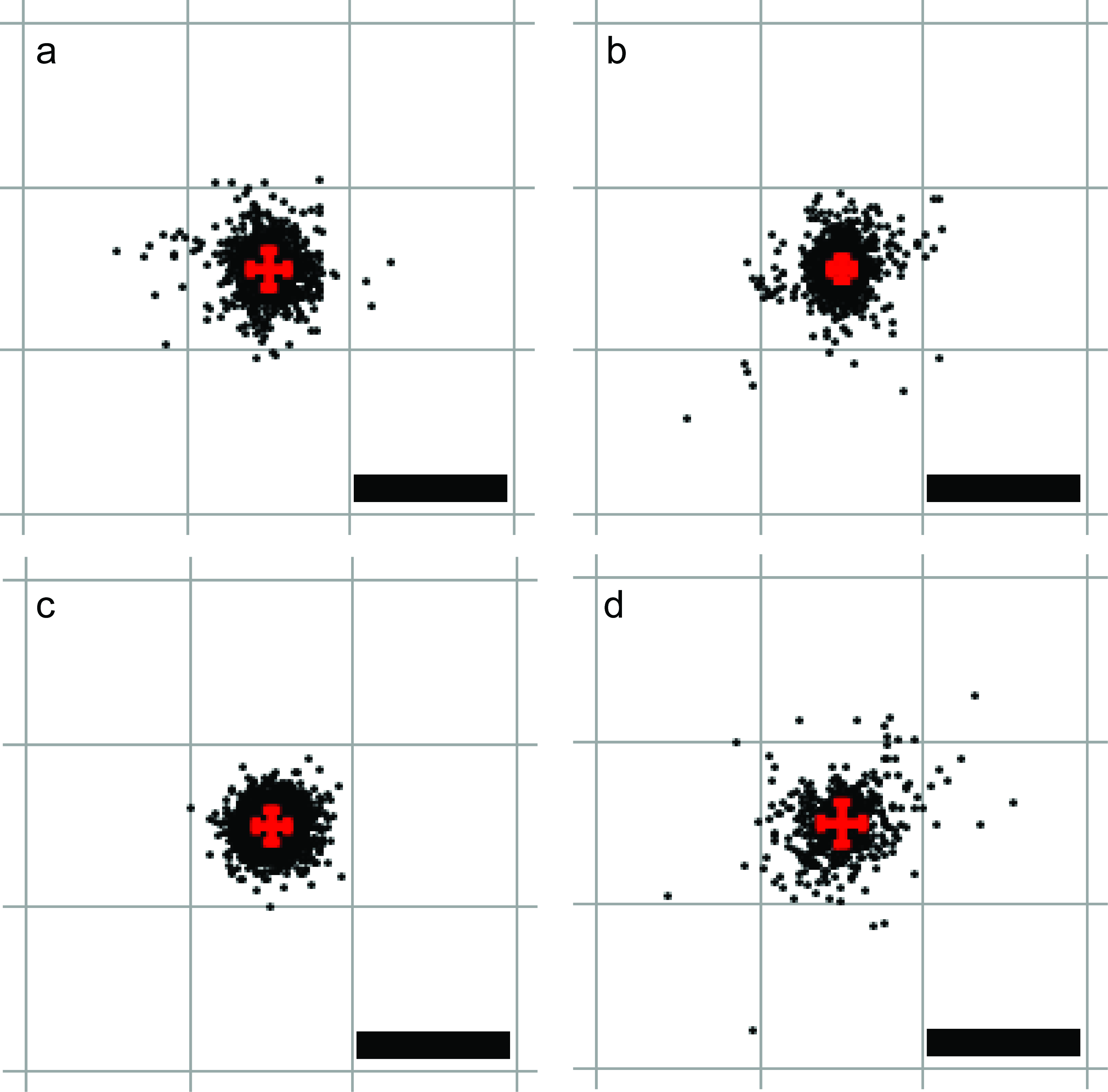

Supplement: Figure S9 — Localization precision of single QD565, QD605, QD655, and QD705 in the QuadView configuration. The localization precision of MC-SPT ín the QuadView configuration with 10 ms image integration was determined by time-lapse imaging and SPT analysis of QDs that had been immobilized on glass. Shown are the determined centroids of N single independent QDs (black points) and the mean centroid (±1 standard deviation; red) of all superimposed centroids, where superpositioning in the center of a projected pixel was done by first subtracting the mean centroid of each single QD respectively. The centroids are displayed on the projected pixel array of the EMCCD (Projected pixel size of ≈108 nm). The localization precision, δr, of the MC-SPT was found to be (a) sAv-QD565: δr≈21 nm (N = 1028), (b) sAv-QD605: δr≈14 nm (N = 1637), (c) sAv-QD655: δr≈15 nm (N = 2689), and (d) sAv-QD705: δr≈28 nm (N = 514). Scale bar is equal to 100 nm. (TIF) [file pone.0048521.s009.tif]

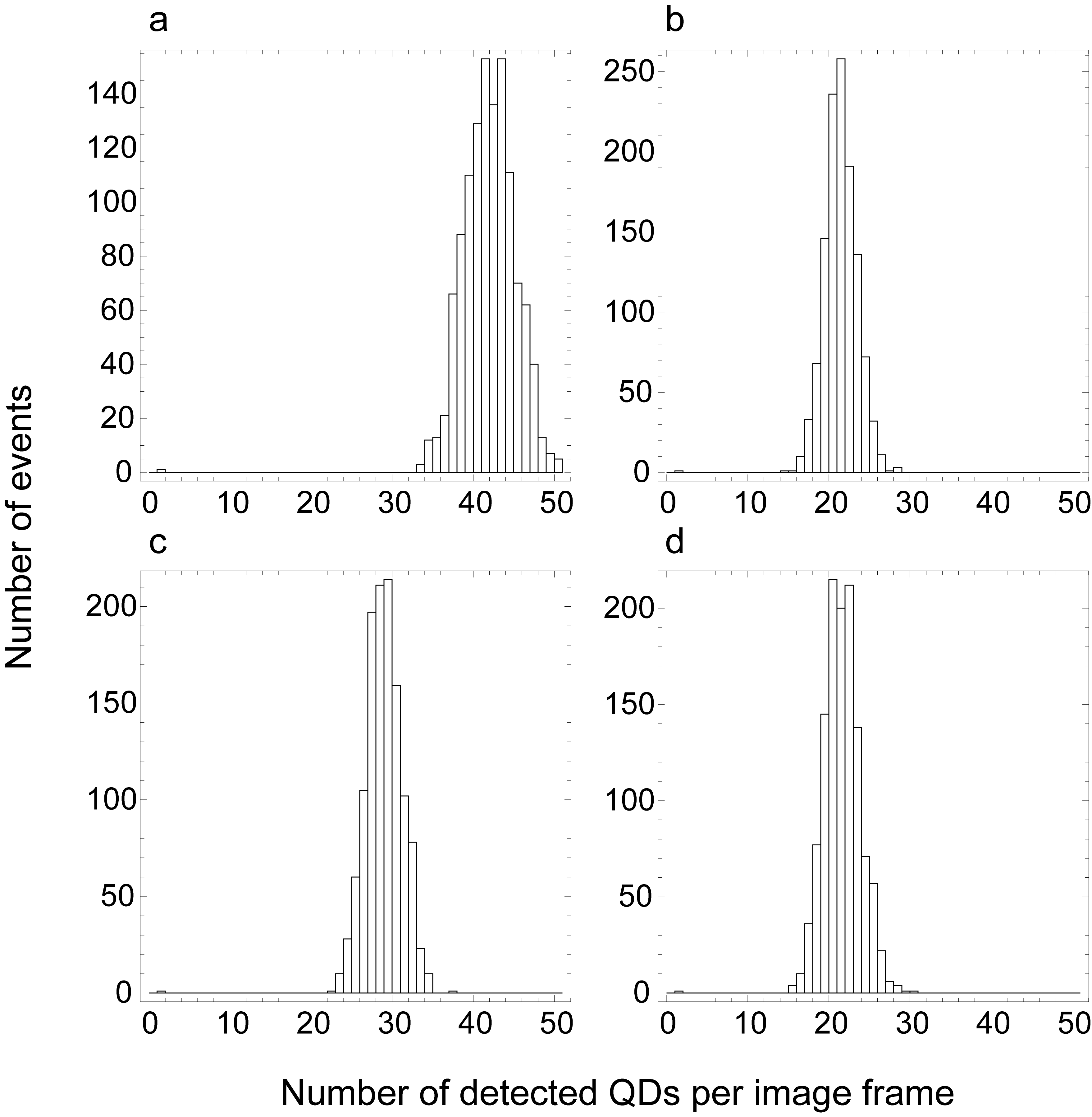

Supplement: Figure S10 — Sample distributions of the number of detected QDs in each image frame in each image channel for the MC-SPT example in Fig. 6 . The mean ±1 SD of the number of detected sAv-QDs in each image channel for the example in Fig. 6 using the SPT analysis as described in the Methods section was for a) sAv-QD565: 42±3, b) sAv-QD605: 21±2, c) sAv-QD655: 28±2, and d) sAv-QD705: 21±2.The total mean ±1 standard deviation of the detected sAv-QDs for all channels were 112±5. In the given example this corresponds to a single QD labeling density of a) sAv-QD565: 0.075±0.005 QDs/µm2, b) sAv-QD605: 0.037±0.004 QDs/µm2, c) sAv-QD655: 0.050±0.004 QDs/µm2, and d) sAv-QD705: 0.037±0.004 QDs/µm2. The overall labeling density for all QDs in all channels was 0.200±0.009 QDs/µm2. (TIF) [file pone.0048521.s010.tif]

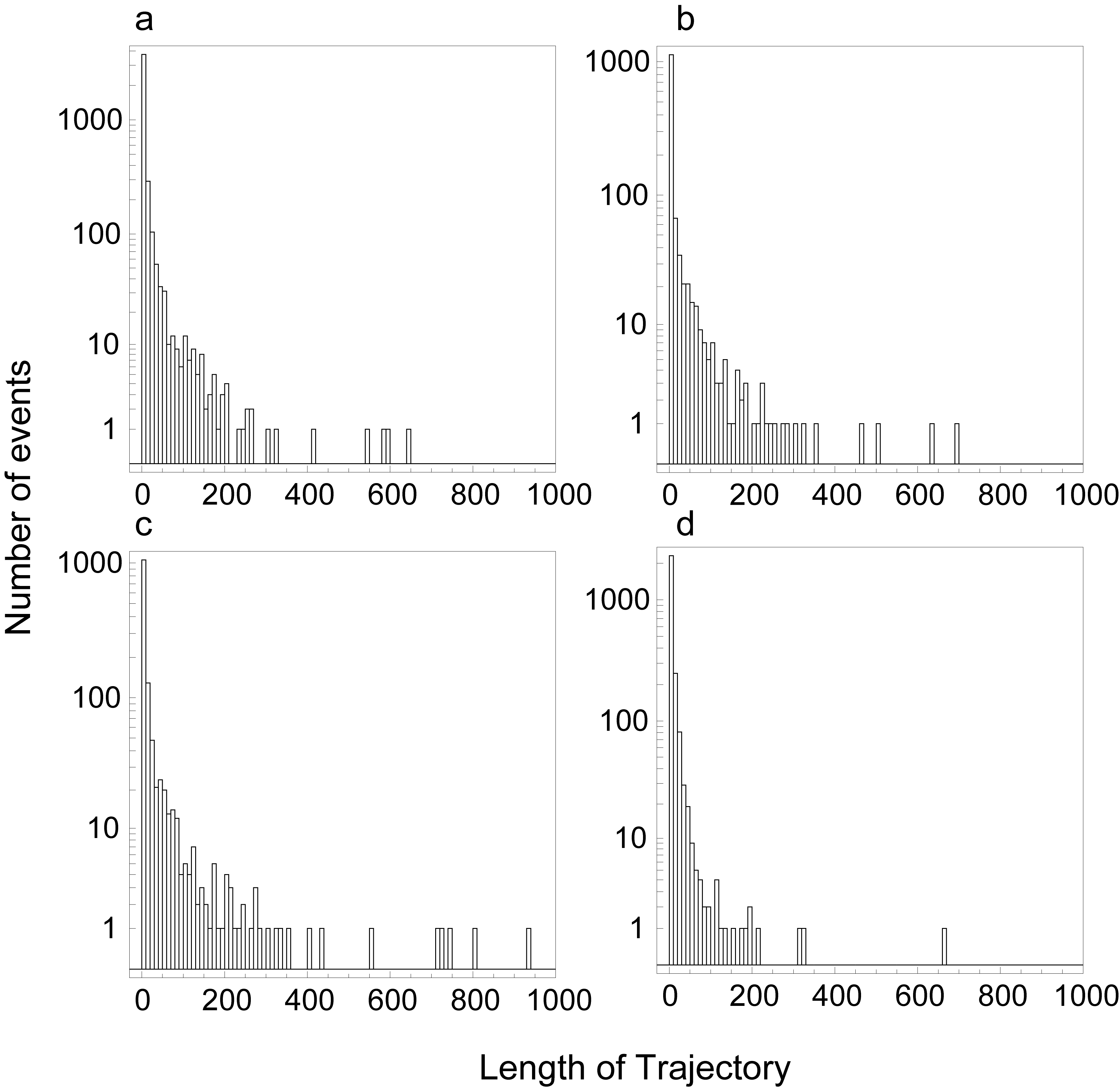

Supplement: Figure S11 — Sample distributions of the length of the detected QD trajectories in each image channel for the MC-SPT example in Fig. 6 . A result of the QD blinking is that SPT with QDs results in many short trajectories such that the number of trajectories is much greater than the number of detected QDs in each image frame. In the given example in Fig. 6, SPT analysis resulted in n trajectories with a length of mean ± SEM. steps of a) sAv-QD565: 9±0.1 (n = 4,356), b) sAv-QD605: 15±0.4 (n = 1,367), c) sAv-QD655: 22±0.6 (n = 1,398), and d) sAv-QD705: 7±0.1 (n = 2,718) steps. Of these trajectories we analyzed only those that were longer than 20 steps. The corresponding numbers for these trajectories were for a) sAv-QD565: 83±6 (n = 289), b) sAv-QD605: 121±16 (n = 142), c) sAv-QD655: 161±20 (n = 162), and d) sAv-QD705: 65±11 (n = 131). The length of the entire image sequence was 1200 image frame acquired at an image acquisition rate of ≈25 Hz corresponding to a total duration of ≈48.3 s. (TIF) [file pone.0048521.s011.tif]

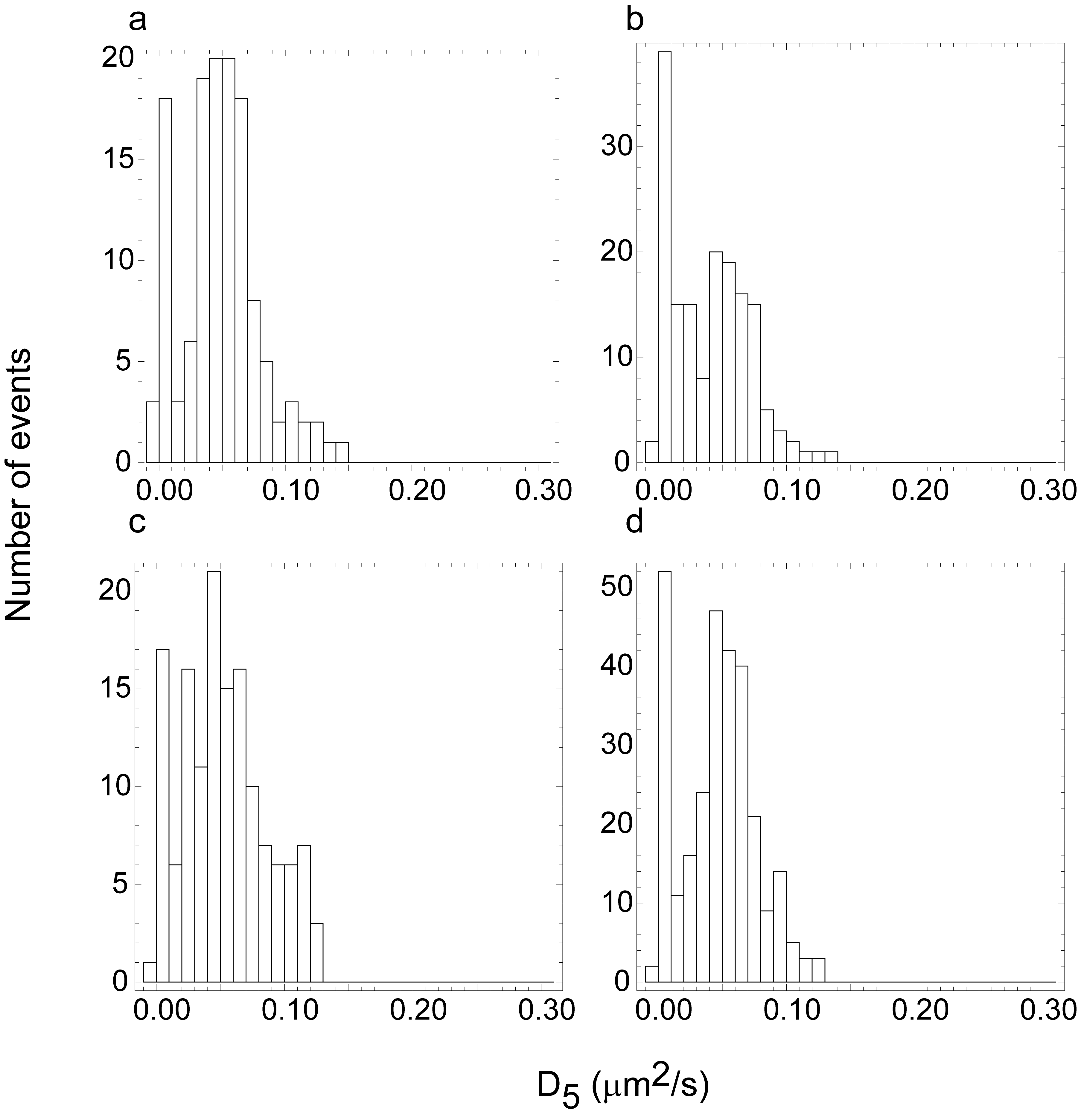

Supplement: Figure S12 — Fitted diffusion coefficients, D5, for biotin-cap-DPPE for each sAv-QD conjugate in each image channel for the MC-SPT example in Figure 6 . The diffusion coefficient, D5, was determined by calculating the MSD curve for each trajectory that were longer than 20 steps, and by curve fitting the initial five points, t < n t <5 t, of each trajectory, as is described in the Methods section. The entire distribution of the fitted values of D5 are shown for all trajectories that were labeled with a) sAv-QD565 (N = 289), b) sAv-QD605 (N = 142), c) sAv-QD655 (N = 162), and d) sAv-QD705 (N = 131). The mean diffusion coefficient, , (± SEM) was for a) sAv-QD565: 0.047±0.002 µm2/s, b) sAv-QD605: 0.052±0.003 µm2/s, c) sAv-QD655: 0.039±0.002 µm2/s, and d) sAv-QD705: 0.049±0.003 µm2/s. (TIF) [file pone.0048521.s012.tif]
