# Supplementary material for: Multi-Color Single Particle Tracking with Quantum Dots
Source: PLoS One. 2012 Nov 14;7(11):e48521. doi: 10.1371/journal.pone.0048521 (PMC3498293; doi:10.1371/journal.pone.0048521)
Supplement: Table S1 — Product specifications of investigated QDs. The reported molar extinction coefficients are those given by the manufacturer. (DOC) [file pone.0048521.s013.doc]

**Supporting Information Table S1.**

| Sample | Product Number | ε (cm^-1^ M^-1^ @ 488 nm) |
| --- | --- | --- |
| sAv-QD525 | Q10151MP | 130,000 |
| sAv-QD565 | Q10151MP | 290,00 |
| sAv-QD585 | Q10151MP | 530,000 |
| sAv-QD605 | Q10101MP | 1,100,000 |
| sAv-QD625 | A10106 | 2,700,000 |
| sAv-QD655 | Q10151MP | 2,900,000 |
| sAv-QD705 | Q10151MP | 3,000,000 |
| AMP-QD800 | Q21371MP | 3,000,000 |
