# Supplementary material for: Multi-Color Single Particle Tracking with Quantum Dots
Source: PLoS One. 2012 Nov 14;7(11):e48521. doi: 10.1371/journal.pone.0048521 (PMC3498293; doi:10.1371/journal.pone.0048521)
Supplement: Table S2 — Quantification of mean intensity, IQDon, and fractional intermittency times, FQDon, of single QDs with 500 LP Emission Filter Microscope Configuration. (DOC) [file pone.0048521.s014.doc]

**Supporting Information Table S2.**

| **QD** | **1 mM DTT** | **Single QDs (n)** | **Sample Pts (n x m)** | **Mean QD Intensity, I_QD_^on^**  **(above Bkgd)**  **(**± **s. e. m.)** | **Fractional On Time,**  **F_QD_^on^** |
| --- | --- | --- | --- | --- | --- |
| sAv-QD525 | - | 265 | 79,500 | 740 ± 30 | 0.81 |
| sAv-QD525 | + | 465 | 139,500 | 600 ± 30 | 0.75 |
| sAv-QD565 | - | 453 | 135,900 | 2310 ± 30 | 0.81 |
| sAv-QD565 | + | 628 | 188,400 | 1780 ± 30 | 0.84 |
| sAv-QD585 | - | 536 | 160,800 | 2950 ± 50 | 0.78 |
| sAv-QD585 | + | 613 | 183,900 | 3020 ± 50 | 0.87 |
| sAv-QD605 | - | 364 | 109,200 | 6240 ± 110 | 0.77 |
| sAv-QD605 | + | 381 | 114,300 | 6420 ±90 | 0.87 |
| sAv-QD625 | - | 645 | 193,500 | 12700 ± 150 | 0.87 |
| sAv-QD625 | + | 649 | 194,700 | 14400 ± 110 | 0.95 |
| sAv-QD655 | - | 377 | 113,100 | 15100 ± 160 | 0.87 |
| sAv-QD655 | + | 400 | 120,000 | 15200 ± 220 | 0.93 |
| sAv-QD705 | - | 316 | 94,800 | 4890 ± 110 | 0.26 |
| sAv-QD705 | + | 510 | 153,000 | 4410 ± 110 | 0.58 |
| Amp-QD800 | - | 115 | 34,500 | 1940 ± 150 | 0.15 |
| Amp-QD800 | + | 149 | 44,700 | 1820 ± 130 | 0.58 |
