# Supplementary material for: Multi-Color Single Particle Tracking with Quantum Dots
Source: PLoS One. 2012 Nov 14;7(11):e48521. doi: 10.1371/journal.pone.0048521 (PMC3498293; doi:10.1371/journal.pone.0048521)
Supplement: Table S3 — Hydrodynamic Radii of QDs. (DOC) [file pone.0048521.s015.doc]

**Supporting Information Table S3.**

| **Sample** | **D (μm^2^/sec)** | **R_H_ (QD) / R_H_ (ms-IgG1)** | **R_H_(nm)** |
| --- | --- | --- | --- |
| Ms-IgG1 | 36.8 ± 3.3 | 1.00 ± 0.13 | 5.6 ± 0.5 |
| AMP-605 | 34.1 ± 2.0 | 1.08 ± 0.12 | 6.0 ± 0.4 |
| NH_2_-PEG-605 | 24.5 ± 1.7 | 1.50 ± 0.17 | 8.4 ± 0.6 |
| sAv-525 | 21.3 ± 1.2 | 1.73 ± 0.18 | 9.7 ± 0.5 |
| sAv-565 | 22.3 ± 1.4 | 1.65 ± 0.18 | 9.3 ± 0.6 |
| sAv-585 | 21.3 ± 1.4 | 1.73 ± 0.19 | 9.7 ± 0.6 |
| sAv-605 | 21.3 ± 1.2 | 1.73 ± 0.18 | 9.7 ± 0.5 |
| sAv-625 | 18.2 ± 1.2 | 2.02 ± 0.23 | 11.3 ± 0.8 |
| sAv-655 | 18.1 ± 1.1 | 2.03 ± 0.22 | 11.4 ± 0.7 |
| sAv-705 | 16.1 ± 3.3 | 2.29 ± 0.51 | 12.8 ± 2.5 |
| AMP-800 | N.D. | N.D. | N.D. |
