# Supplementary material for: Multi-Color Single Particle Tracking with Quantum Dots
Source: PLoS One. 2012 Nov 14;7(11):e48521. doi: 10.1371/journal.pone.0048521 (PMC3498293; doi:10.1371/journal.pone.0048521)
Supplement: Table S4 — Quantification of fluorescence intensities, IQDon, and fractional intermittency times, FQDon, of single QDs with QuadView microscope filter configuration. (DOC) [file pone.0048521.s016.doc]

**Supporting Information Table S4.**

| **QD** | **Single QDs (n)** | **Sample Pts (n x m)** | **Mean QD Intensity, I_QD_^on^**  **(above Bkgd)**  **(**± s**. e. m.)** | **Fractional Intermittency Time, F_QD_^on^** |
| --- | --- | --- | --- | --- |
| sAv-QD565 | 211 | 38,100 | 1370 ± 70 | 0.96 |
| sAv-QD605 | 250 | 75,000 | 5820 ± 210 | 0.99 |
| sAv-QD655 | 78 | 23,400 | 3460 ± 150 | 0.95 |
| sAv-QD705 | 43 | 12,900 | 1750 ± 150 | 0.21 |
