# Supplementary material for: Multi-Color Single Particle Tracking with Quantum Dots
Source: PLoS One. 2012 Nov 14;7(11):e48521. doi: 10.1371/journal.pone.0048521 (PMC3498293; doi:10.1371/journal.pone.0048521)
Supplement: Table S5 — Localization precision of single QD imaging. (DOC) [file pone.0048521.s017.doc]

**Supporting Information Table S5.**

| **QD** | **500LP Microscope Configuration**  **t_acquisition_ = 5 ms** | | **QuadView Microscope Configuration**  **t_acquisition_ = 10 ms** | |
| --- | --- | --- | --- | --- |
|  | **Sample Pts** | **Localization Precision**  **δr=(δx^2^+δy^2^)^1/2^ (nm)** | **Sample Pts** | **Localization Precision**  **δr=(δx^2^+δy^2^)^1/2^ (nm)** |
| sAv-QD525 | 254 | 30 | - | - |
| sAv-QD565 | 1,441 | 22 | 1,028 | 21 |
| sAv-QD585 | 1,187 | 22 | - | - |
| sAv-QD605 | 8,598 | 14 | 1,637 | 14 |
| sAv-QD625 | 1,155 | 16 | - | - |
| sAv-QD655 | 2,979 | 14 | 2,689 | 15 |
| sAv-QD705 | 427 | 16 | 514 | 28 |
| Amp-QD800 | 337 | 23 | - | - |
