# Supplementary material for: Multi-Color Single Particle Tracking with Quantum Dots
Source: PLoS One. 2012 Nov 14;7(11):e48521. doi: 10.1371/journal.pone.0048521 (PMC3498293; doi:10.1371/journal.pone.0048521)
Supplement: Table S6 — Summary of MC-SPT results for four color tracking of biotin-cap-DPPE labeled with each type of sAv-QD. (DOC) [file pone.0048521.s018.doc]

**Supporting Information Table S6.**

|  | Mean ± s.t.d.  QDs per image frame | n  (Trajectories > 20 steps) | Mean ± s.e.m. Trajectory Length (steps) | Mean ± s.e.m.  <D_5_> (μm^2^/s) |
| --- | --- | --- | --- | --- |
| sAv-QD565 | 42±3 | 289 | 83 ± 6 | 0.047±0.002 |
| sAv-QD605 | 21±2 | 142 | 121±16 | 0.052±0.003 |
| sAv-QD655 | 28±2 | 162 | 161±20 | 0.039±0.002 |
| sAv-QD705 | 21±2 | 131 | 65±11 | 0.049±0.003 |
