# Supplementary material for: Multi-Color Single Particle Tracking with Quantum Dots
Source: PLoS One. 2012 Nov 14;7(11):e48521. doi: 10.1371/journal.pone.0048521 (PMC3498293; doi:10.1371/journal.pone.0048521)
Supplement: Table S7 — p-values for non-parametric two-sided Kolmogorov-Smirnov test of the diffusion coefficient of biotin-cap-DPPE in live MEFs. White shade = Statistical significant difference at confidence level of α ≤ 0.05. Gray shade = No statistical significant difference at confidence level of α ≤ 0.05. (DOC) [file pone.0048521.s019.doc]

**Supporting Information Table S7.**

| p; H_0_: μ_1_ - μ_2_ = 0 | sAv-QD565  D_5_  n=289 | sAv-QD605  D_5_  n=142 | sAv-QD655  D_5_  n=162 | sAv-QD705  D_5_  n=131 |
| --- | --- | --- | --- | --- |
| sAv-QD565  D_5_  n=289 |  | 0.3139 | 0.0078 | 0.8031 |
| sAv-QD605  D_5_  n=142 | 0.03139 |  | 0.0085 | 0.3996 |
| sAv-QD655  D_5_  n=162 | 0.0078 | 0.0085 |  | 0.0018 |
| sAv-QD705  D_5_  n=131 | 0.8031 | 0.3996 | 0.0018 |  |
